# Genetic Dissection of Yield and Its Component Traits Using High-Density Composite Map of Wheat Chromosome 3A: Bridging Gaps between QTLs and Underlying Genes

**DOI:** 10.1371/journal.pone.0070526

**Published:** 2013-07-24

**Authors:** Sachin Rustgi, Mustafa N. Shafqat, Neeraj Kumar, P. Stephen Baenziger, M. Liakat Ali, Ismail Dweikat, B. Todd Campbell, Kulvinder Singh Gill

**Affiliations:** 1 Department of Crop and Soil Sciences, Washington State University, Pullman, Washington, United States of America; 2 Department of Biosciences, COMSATS Institute of Information Technology, Islamabad, Pakistan; 3 Department of Agronomy and Horticulture, University of Nebraska-Lincoln, Lincoln, Nebraska, United States of America; 4 Agricultural Research Service, Coastal Plains Soil, Water, and Plant Research Center, Florence, South Carolina, United States of America; Nanjing Forestry University, China

## Abstract

Earlier we identified wheat (*Triticum aestivum* L.) chromosome 3A as a major determinant of grain yield and its component traits. In the present study, a high-density genetic linkage map of 81 chromosome 3A-specific markers was developed to increase the precision of previously identified yield component QTLs, and to map QTLs for biomass-related traits. Many of the previously identified QTLs for yield and its component traits were confirmed and were localized to narrower intervals. Four novel QTLs one each for shoot biomass (*Xcfa2262-Xbcd366*), total biomass (*wPt2740-Xcfa2076*), kernels/spike (KPS) (*Xwmc664-Xbarc67*), and *Pseudocercosporella* induced lodging (*Ps*IL) were also detected. The major QTLs identified for grain yield (GY), KPS, grain volume weight (GVWT) and spikes per square meter (SPSM) respectively explained 23.2%, 24.2%, 20.5% and 20.2% of the phenotypic variation. Comparison of the genetic map with the integrated physical map allowed estimation of recombination frequency in the regions of interest and suggested that QTLs for grain yield detected in the marker intervals *Xcdo549-Xbarc310* and *Xpsp3047-Xbarc356* reside in the high-recombination regions, thus should be amenable to map-based cloning. On the other hand, QTLs for KPS and SPSM flanked by markers *Xwmc664* and *Xwmc489* mapped in the low-recombination region thus are not suitable for map-based cloning. Comparisons with the rice (*Oryza sativa* L.) genomic DNA sequence identified 11 candidate genes (CGs) for yield and yield related QTLs of which chromosomal location of two (*CKX2* and *GID2-like*) was confirmed using wheat aneuploids. This study provides necessary information to perform high-resolution mapping for map-based cloning and for CG-based cloning of yield QTLs.

## Introduction

Grain yield is one of the most important targets for agricultural research, as well as for sustaining human and livestock populations on this planet. In addition to securing global food resources, grain yield is also important for understanding history of crop domestication [1]. Grain yield in wheat (*Triticum aestivum* L.) can be largely partitioned into three major components: kernel number per spike, kernel weight, and spikes or reproductive tillers per unit area. These traits are affected by other traits including anthesis date, plant biomass, and plant height. Each yield component trait is controlled by multiple loci following complex genetic interactions [2], [3]. Some of the component traits, such as grain weight and plant height, are more stably inherited compared to others including grain number and root/shoot biomass that are more variable in nature and show stronger genotype by environment interactions [4].

QTLs for yield or its component traits have been mapped on wheat homoeologous groups 2, 3, 5 and 7 chromosomes [4] of which QTLs on chromosome 3A are by far the best characterized and are the most reproducibly detected [5]. Initial identification of chromosome 3A as a major determinant of grain yield came from the studies conducted on a full set of chromosome substitution lines developed from two historically important hard red winter wheat cultivars, Wichita (WI) and Cheyenne (CNN). WI chromosomes 3A and 6A when substituted for the corresponding CNN chromosomes showed 19% and 14% yield advantage, respectively over CNN [6], [7]. The reciprocal substitution lines for these chromosomes respectively showed 17% and 23% reduction in grain yield in comparison with WI.

Initial mapping of QTLs to sub-chromosomal region(s) on 3A was performed relative to 13 DNA markers using a set of 50 recombinant inbred chromosome lines (RICLs) [8], [9]. QTLs for various yield component traits including anthesis date, plant height, kernel number per spike, 1000-kernel weight and spike number per square meter were identified but not for the grain yield itself. The complex nature of the trait, smaller population size, low map resolution, and strong genotype by environment interaction probably prevented detection of QTL(s) for grain yield. This study was further refined by increasing the population size to 95 RICLs (including the original set of 50 RICLs) and the molecular marker number to 20 [10]. Four major chromosomal regions harboring QTLs for grain yield and various component traits were identified. In a follow-up study, the number of 3A-specific markers was increased to 41 that further improved the precision of QTL analysis [11]. The population size was then increased to 223 RICLs by mapping 32 chromosome 3A-specific markers on 128 additional RICLs generated by doubled haploid (DH) method [12]. The complete set of 223 RICLs was evaluated for yield and its component traits for six environment years from 2005 to 2007, and the data was used for QTL analysis. In addition to confirming many of the previously identified QTLs and improving resolution in a few cases, the study showed that the two RICL development methods have similar power of QTL detection, thus can be interchangeably used.

Most of the QTLs detected on chromosome 3A mainly mapped to two regions, namely region 1 and region 2, respectively overlapping with the gene-rich regions 3S0.9 (46 Mb; 1.1 Mb/cM) demarcated by the deletion breakpoint of 3BS-3, and 3S0.8 (25 Mb; 0.58 Mb/cM) by 3BS-8 and 3AS-3 [10]–[13]. Localization of these QTLs was later reconfirmed by the use of a mirror population WI(CNN3A) of 90 RICLs [14]. As in previous studies, QTLs for various yield components co-localized to the two chromosomal regions. To determine if the co-localization is a consequence of tight linkage or pleiotropy, it is important to increase marker density and map resolution.

Objectives of the present study were to: i) increase the marker density of the 3A map to localize QTLs to narrower intervals; ii) identify QTLs for biomass related traits including root and shoot biomass in wheat, and iii) identify rice genomic regions corresponding with the QTL-containing regions of wheat to identify additional markers and genes putatively underlying the QTLs. Furthermore, we intended to compare number and quality of QTLs identified from greenhouse evaluation with that from multi-location field trials, and effect of increasing the marker number with plant number on the efficacy of QTL detection and resolution.

## Materials and Methods

### Plant Material and Growth Conditions

Genetic analyses of agronomically important traits were performed using a set of 95 chromosome 3A specific recombinant inbred chromosome lines (3A-RICLs), derived from a cross between wheat cultivar Cheyenne (CNN) and its substitution lines CNN(WI3A), carrying chromosome 3A from wheat cultivar Wichita (WI). Procedure for the development of the RICL population was described elsewhere [8], [9], [15]. An extended set of 223 RICLs including the above 95 RICLs was also used for genotyping and map construction. The objective of using the extended population was to study the effect of increasing population size on the map resolution. The procedure for the development of the 128 additional RICLs was described elsewhere [12].

Phenotypic data on the 95 RICLs along with five check cultivars (Goodstreak, Pronghorn, Arapahoe, Jagger and WI) were gathered in two different ways: (i) in field, at three different locations (Lincoln, Mead and Sidney, NE) for seven years including 1999 to 2001 and 2005 to 2008, and (ii) in the greenhouse. In 1999, 2007 and 2008, the phenotypic data was collected respectively at Lincoln (NE), Mead (NE) and Pullman (WA), and in 2006 at Lincoln and Mead. In the field, phenotypic data were collected for grain yield (GY), spike/square meter (SPSM), kernels/spike (KPS), grain volume weight (GVWT), 1000-kernel weight (TKW), plant height (PH), and heading date (HD), as described by Campbell et al. [10]. The data on *Pseudocercosporella* induced lodging (*Ps*IL), also known as eyespot or strabreaker foot rot, was recorded on an arbitrary scale of 1 to 10 (where 1 represents <10% lodging and 10 represents complete lodging) at Pullman in 2008. The disease is caused by *P. herpotrichoides* (Fron.) Deighton, which results in weakening of the stem base eventually leading to the lodging of the infested plants. Briefly, each entry was grown in a four-row plot of 2.4 m length and 30 cm row-spacing with four replications following a randomized complete block design (RCBD) in 1999, 2005–2007, and 2008, and following incomplete block design (ICBD) in 2000 and 2001. In 2005 Lincoln trial, data were not recorded on GY, SPSM, KPS and TKW because the field was infested with the soil borne wheat mosaic virus. Similarly in 2008, data could only be recorded for plant height and *Ps*IL.

For the greenhouse study, seeds of the 95 RICLs along with parents were planted in vermiculite medium and seedlings at the two-leaf stage were vernalized at 4°C for eight weeks. Subsequently, these seedlings were transplanted in plastic pots each lined with polyethylene bags containing two-kg of acid-washed course sand. For drainage small holes were punched in the polyethylene bags. Each pot was supplied with 250 ml of Hoagland solution adjusted to pH 5.7 per day to meet the nutritional and water requirements of plants throughout the study. The plants in the greenhouse were grown at the day/night temperatures of 25/18°C with 16 hrs of supplemental light. The study was conducted using a randomized complete block design with three replications of each entry. Data on various phenotypic traits were recorded at different developmental stages. Plant height (PH) was recorded in cm by measuring the tallest tiller from ground to the tip of the spike excluding awns, heading date (HD) was measured in number of days from sowing to emergence of at least three spikes from the leaves. The 1000-kernel weight (TKW) in grams (g) was computed from the weight of all kernels harvested per plant. Data on total number of kernels per plant, their weight, and total number of spikes per plant were used to calculate kernels per spike (KPS). Seed weight per spike (SWPS) was calculated by dividing total seed weight by total number of spikes.

For biomass related traits, each plant was harvested at maturity and dissected into three parts: roots, spikes, and everything else. The plant material was dried at 50°C for three days before recording weight. Shoot biomass (SB) included weight of all above ground plant parts excluding roots. For root biomass (RB), the below ground plant parts with sand were placed on a sieve of an appropriate mesh size that allowed collection of roots without sand particles. The total biomass was calculated from the sum of root and shoot biomass recorded for each plant. One-way analysis of variance was performed and the least significant difference (P<0.05) was used to partition significant difference in means of various traits. All statistics were performed using SAS statistical software version 8.1 (SAS Institute, North Carolina).

### Ethics Statement

No specific permissions were required for the field locations/activities because UNL is a land-grant university, and the Department of Agronomy & Horticulture operates farms at several different locations in Nebraska including farms used in the present study at Lincoln, Mead and Sidney. These farms are dedicated to research activities, where trials were performed under the vigilance of respective farm managers strictly following the guidelines formulated by the Department of Agronomy & Horticulture. WSU is also a land-grant university and the Department of Crop & Soil Sciences operates three farms at Pullman including the Spillman Agronomy Farm, where the research trial was performed in 2008. This trial was performed under the supervision of the farm manager following the guidelines set by the Department of Crop & Soil Sciences.

### DNA Marker Analysis and Map Construction

Available molecular markers including restriction fragment length polymorphism (RFLP), simple sequence repeat (SSR), sequence-tagged microsatellite (STM) and diversity array technology (DArT) were used to maximize the number of markers on the 3A map. For molecular marker analysis, genomic DNA was extracted from the leaves of 3 weeks-old seedlings following modified CTAB method as described by Sandhu et al. [16]. For RFLP analysis, a total of 179 probes specific to wheat group 3 chromosomes was selected and used following the method described in Gill et al. [17]. The 179 RFLP probes included 36 ESTs (http://wheat.pw.usda.gov) and 143 cDNA and/or *Pst*I digested genomic DNA probes derived from *Aegilops tauschii* Coss., *Hordeum vulgare* L., *Avena sativa* L. and *T. aestivum* L. (for probe details, see ref. [11]). Similarly for SSR and STM analysis a total of 110 chromosome 3A specific markers (including 90 SSRs and 20 STMs) were used for polymorphism survey following the PCR conditions described in Shah et al. [18]. Primer sequences of the SSR markers with prefixes ‘Xbarc’, ‘Xwmc’, and ‘Xcfa’ were retrieved from the GrainGenes (http://wheat.pw.usda.gov/GG2/index.shtml) while sequences of ‘Xgwm’ primers were obtained from Röder et al. [19], ‘Xpsp’ from Stephenson et al. [20], ‘Xhbg’ from Torada et al. [21] and STM primers sequence from Hayden et al. [22]. All primer pairs were synthesized from Operon Technologies (Huntsville, Alabama). Polymorphism survey for DArT markers was performed using wheat *Pst*I (*Taq*I) v2.6 discovery array and genotyping using the population specific array by Triticarte Pty. Ltd. (Australia). The maps were constructed using MAPMAKER/EXP version 3.0b [23] with the Kosambi mapping function [24].

### QTL Analysis

Composite interval mapping (CIM) was conducted by QTL Cartographer version 2.5 [25] using mapping information and phenotypic data recorded for each trait. For CIM, forward and backward stepwise regressions were performed to select five markers as cofactors, and the analysis was conducted using ‘model 6’ with a moving window size of 10 cM. At each interval, the significance of the QTL-trait association was tested by the likelihood ratio statistics (LRS) [26]. For each trait, a significant threshold was estimated by 1000-permutations at P<0.05. However, an average LOD score of 2.0 was used to declare the presence of a putative QTL. For all traits, QTL analyses were carried out using RICL means for individual environments, as well as, the pooled data averaged over all environments. Phenotypic variation (r^2^) explained by a QTL was calculated at the peak LOD value of the plot. Confidence intervals (CI) were estimated by marking positions ±1 LOD from the peak [27]. QTLs within 1.0 cM distance or with overlapping confidence intervals were considered single QTL.

### Integrated Physical Map

A deletion breakpoint-based physical map of wheat homoeologous group 3 chromosomes was previously constructed [11], [28]. Starting with the physical map of Dilbirligi et al. [11] as a base, markers and genes were added to the physical map after an extensive survey of the literature. QTLs detected for a number of agronomically important traits in different studies were also assigned to their respective locations on the integrated physical map.

### Wheat-rice Comparison

To identify candidate genes and additional markers underlying QTLs of interest and to determine the linear order of markers within each deletion-bin, wheat chromosome 3A-specific sequences (table S1) were compared with the rice genomic DNA sequences available at various websites (http://wheat.pw.usda.gov, http://ncbi.nlm.nih.gov, http://gramene.org/, and http://www.tigr.org). The DNA sequence comparisons were made using BLASTn option at an e-value<l × 10^−19^ and score >100. Initially, chromosome 3A specific sequences were compared to rice chromosome 1 BAC/PAC sequences to demarcate collinear regions underlying specific QTLs on the rice chromosome. The identified rice BAC/PAC sequences were then compared to wheat 3A and 3B BAC end sequences (BES) in order to identify additional markers for each QTL interval. The identified rice BAC/PAC sequences underlying the QTL were manually curated to identify candidate genes. Protein sequences of the identified genes were compared against wheat ESTs using tBLASTn searches to identify wheat orthologues of the candidate genes (e-value<l×10^−25^).

### High-resolution Melt (HRM) Curve Analysis

Physical mapping of the candidate genes was attempted by HRM curve analyses on Roche LightCycler® 480 (Roche Inc., USA). The following PCR primers were used to amplify wheat orthologues of *GID2-like* (F: 5′-GAGCACGAGCACAGGTTGTT-3′; R: 5′-GTGGTGATGTCGTGGATGCT-3′) and *CKX2* (F: 5′-CGTGCAGGACGTGTCGTA-3′; R: 5′-GTTCCGGTTCATGGGGTAG-3′) genes. The reaction mixture consisted of 30 ng DNA, 5 µM of each primer, 1.5 mM MgCl_2_, and 10 µL of the master mix (2X) provided with the LightCycler® 480 high resolution melting master kit (Roche Diagnostics GmbH, Germany), in a total volume of 20 µl. A negative control containing all reagents except genomic DNA was included in each run. The PCR profile used was: 10 min at 95°C, followed by 45 cycles of 30 s at 95°C, 30 s at 60°C and 45 s at 72°C, and 10 min at 72°C for the final extension. The melt curve analysis was performed upon completion of the amplification cycles by ramping up the temperature from 65°C to 97°C, rising by 0.02°C/s with continuous acquisition of fluorescence. The automated genotype calling software (LightCycler® 480, version 1.5) was used to determine the genotypes of individual lines.

## Result and Discussion

### Greenhouse Evaluation of Traits

In view of accessing reliability and the applied value of phenotypic measurements made on the complex traits under the controlled environment, and to evaluate 95 RICLs 3A-population for plant biomass-related traits, data were recorded on the following traits: RB, SB, TB, PH, HD, TKW, SWPS and KPS. The one-way ANOVA, trait correlations, and subsequently QTL analyses were then conducted.

The results of one-way ANOVA suggested that RICLs were significantly different for all studied traits with a P value of <0.001 to 0.05. The only exception in the greenhouse was TKW, which was not significantly different (P value = 0.05). The coefficient of variation (CV), which was used to measure reliability index for various traits, ranged from 4.5 for HD to 29.3% for RB. The traits other than HD that had CV values less than 10% are PH and TKW. The CVs for the remaining traits were close to 15%. Our previous results showed that the traits with CV <15% can be used reliably for genetic studies [10]. Most of the traits scored in this study had CV values <15% with an exception of RB that had 29% CV. The high CV value for RB makes it difficult to characterize this trait, and can be attributed to the loss of root biomass due to microbial oxidation during the later stages of the experiment, although the extent of microbial activities are expected to be minimum under the sand culture. This suggests that the sand culture method is an efficient way of keeping the environmental variations to minimum for most of the studied traits (except for RB), thus can be used to record complex biological traits.

When correlations were studied among traits, shoot biomass showed positive correlations with all traits except KPS (table S2). In comparison with CNN a reduction in root and shoot biomass of CNN(WI3A) was observed, although differences between WI and CNN were non-significant. This observation suggested presence of modifier(s) in the WI background that negatively influenced the trait (table S3).

### Integrated Genetic Linkage Map

Previously we reported chromosome 3A genetic linkage map of 41 markers including 26 RFLPs and 15 SSRs [11]. In the present study, we report development of two genetic linkage maps. The map using the same set of 95 RICLs has 81 molecular markers including 31 SSRs, 1 STM, 25 RFLPs and 24 DArT markers. Another map with 41 molecular markers, including 32 SSRs and 9 RFLPs, was developed using an extended set of 223 RICLs - consisting of 95 previously used and 128 newly developed lines (for details, see ref. [12]). The polymorphism survey of the two parents of the RICL-3A population with 90 chromosome 3A-specific SSRs identified 31 (34.4% polymorphism) polymorphic markers. Similar analysis of 20 STMs identified only one (5% polymorphism) polymorphic marker. Polymorphism analysis using wheat *Pst*I (*Taq*I) v2.6 discovery DArT array identified 56 (36.3%) of the 154 3A specific markers to be polymorphic between the two parents. These results showed that both DArT and SSR markers were equally efficient in detecting polymorphism in wheat whereas STMs were significantly less efficient. Testing of 142 RFLP markers for polymorphism identified 29 (20.42%) polymorphic markers with 11 restriction enzymes.

**Figure 1:**
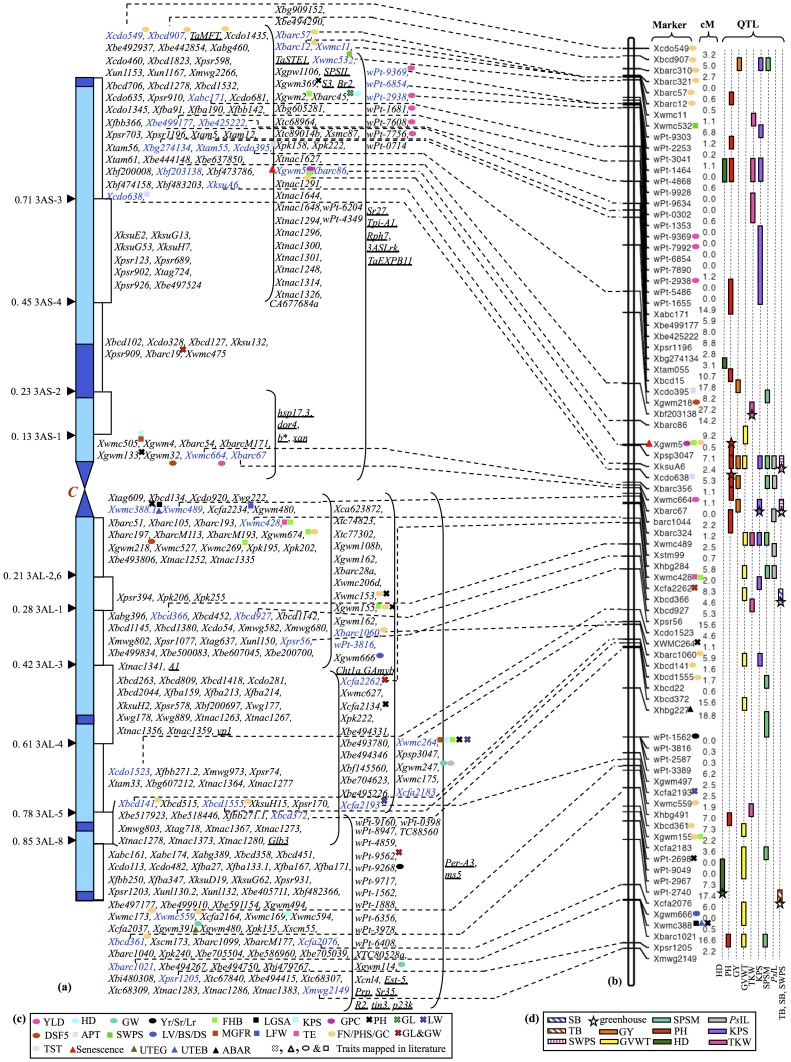
Cytogenetic-ladder map of wheat chromosome 3A showing locations of genes and/or QTLs influencing a number of agronomically important traits. Markers showing connection between genetic and cytogenetic maps are highlighted in blue on the cytogenetic map. (A) Consensus cytogenetic map of chromosome 3A developed by integrating information for additional markers, genes (underlined) and QTLs on the reference map [11]. (B) Integrated genetic linkage map developed by incorporating SSR, STM and DArT markers on the RFLP skeleton map. The map was used to demarcate locations of consistent QTLs detected in the present study and to depict locations of QTLs detected for a number of agronomical traits in the published literature. (C) List of traits and symbols used to demarcate locations of QTLs published elsewhere. YLD = yield; HD = heading date; GW = grain weight; Yr/Sr/Lr = yellow, stem and leaf rust resistance; FHB = *Fusarium* head blight resistance; LGSA = leaf glutamine synthetase activity; KPS = kernels per spike; GPC = grain protein content; PH = plant height; GL = grain length; LW = leaf waxiness; DSF = domestication syndrome factor; APT = adult plant type; SWPS = seed weight per spike; LV/BS/DS = loaf volume, bread score and dough score; MGFR = mean grain filling rate; LFW = leaf fresh weight; TE = transpiration efficiency; FN/PHS/GC = falling number, preharvest sprouting tolerance and grain color; GL&GW = grain length and grain width; GVWT = grain volume weight; PGMS = percent greenness at maximum senescence; UTEB = Phosphorus utilization efficiencies based on biomass yield; UTEG = Phosphorus utilization efficiencies based on grain yield; ABAR = ABA responsiveness. (D) List of traits mapped during the present study (see M&M for details). Traits mapped using data recorded in glasshouse are marked with a star.

The map with 81 markers was prepared by using the previously mapped 25 RFLPs (excluding *Xfba347*) and 14 SSRs (excluding *Xwmc264*) as anchors to assign newly identified 74 polymorphic markers. Of these 74 markers, 56 could be localized to the 3A map increasing the marker number to 81 and the map length from 213.8 cM [11] to 354.7 cM with an average distance of 4.37 cM between the markers (figure 1). Similarly, the map with 41 markers was developed by integrating 18 additional SSRs markers to 14 previously mapped SSRs and 9 RFLPs (figure S1).

Of the 81 markers, six (*Xbarc310*, *Xbarc321*, *Xbarc57*, *Xbarc12*, *Xwmc11*, and *Xwmc532*) showed distorted segregation, skewed in favor of the CNN(WI3A) parent. All of these markers are present at the tip of the short arm suggesting that the distal end of the short arm from WI transmits preferentially over that from CNN. Another explanation for this distorted segregation could be the presence of the earliness QTL in the region and unintentional selection of lines based on their maturity times during the RICL development process.

Both the RFLP and SSR markers were uniformly distributed throughout the map, however, the DArT markers showed severe clustering (figure 1) [29]. The DArT markers formed three clusters on the 3A map with many of the markers showed perfect or tight linkage suggesting that either these markers represent the same locus or are very tightly linked. For all practical purposes, the mapped DArT markers in this study served as three unique loci. Clustering of the DArT markers with no interspersion with the other marker types suggested that these markers might actually be representing part of the genome different from that represented by RFLPs and SSRs. If not all, most of the RFLP markers represent the genic fraction of the wheat genome and the SSRs were interspersed with the RFLP markers. Furthermore, very few of the DArT markers showed associations with any of the QTLs identified in this or the previous studies (see figure 1). Taken together, these results suggested that although DArT markers showed high-level of polymorphism they generated redundant information and most likely represented repetitive fraction of the genome, thus are less applicable for gene mapping studies.

In general, the order of the markers on the current map is in conformity with that on the maps developed by Somers et al. [30] and Song et al. [31]. Except for the DArT markers, distribution of other markers is uniform. There were five regions with genetic distance longer than 16 cM. These regions could either represent highly recombinogenic regions or regions with low polymorphism between the two parents. Although low levels of nucleotide diversity in the proximal regions of chromosome 3A has been reported previously [32], it appears to be a less likely explanation because the five regions with more than 16 cM genetic distances were present in the distal halves of the chromosome. As the distal chromosomal regions have been shown to be highly recombinogenic [13], longer than 16 cM genetic distance in the five regions can thus be attributed to higher recombination rates.

### QTL Analysis

Starting with the studies implicating chromosome 3A in determining yield characteristics [6], [7], [33], our earlier QTL mapping studies facilitated both physical and genetic demarcation of chromosomal regions harboring genes/QTLs for yield and its component traits [8]–[12]. QTL analysis during the present study was conducted using the genetic mapping information and phenotypic data recorded on 95 RICLs-3A mapping population for 7 agronomic traits (including 2 growth related and 5 yield contributing traits) over 12 to 14 environments. In view of studying the effect of ambient environmental conditions on the detection of QTLs, we also recorded data for eight agronomic traits in greenhouse including three biomass related traits for which recording of data in field is practically challenging. The results of QTL analyses are summarized as follows.

### QTL Analysis Using Greenhouse Data

Ten QTLs were detected for seven of the eight traits that were scored in the greenhouse (table 1 and figure 1). The LOD scores ranged from 2.2 for TB and TKW to 4.7 for PH. The range of phenotypic variation explained (PVE) was 9.12% for KPS to 15.22% for SWPS, and the additive effects were negative for all QTLs except that for TB.

**Table 1:** List of QTLs detected on chromosome 3A in the glasshouse study conducted at Pullman, WA.

Trait	QTL interval	cM	LOD	R^2^	Additive effect	Position
HD	*wPt_2698-wPt_2740*	7.4	3.2	18.36	−1.71	307.1
PH	*Xgwm5-Xpsp3047*	0.5	4.7	19.4	−3.61	156.8
	*Xpsp3047-XksuA6* *	7.1	4.2	17.14	−3.46	159.7
	*Xcdo638-Xbarc356* *	5.3	4.5	17.58	−3.53	171.4
TKW	*Xgwm218-Xbf20313*	27.8	2.2	14.73	−0.89	105.9
KPS	*Xwmc664-Xbarc67*	1.1	2.4	9.12	−1.33	173.9
SB	*Xcfa2262-Xbcd366*	8.3	3.4	13.52	−1.63	189.3
TB	*wPt_2740-Xcfa2076*	17.7	2.2	18.25	7.15	316.9
SWPS	*Xpsp3047– XksuA6*	7.1	2.6	11.81	−0.06	157.6
	*Xwmc664-Xbarc67*	1.1	3.5	15.22	−0.07	173.9

HD = heading date, PH = plant height, TKW = 1000-kernal weight, KPS = kernels per spike, SB = shoot biomass, TB = total biomass, SWPS = seed weight per spike.

* QTLs detected in field data analysis.

A QTL was detected each for SB (*Xcfa2262-Xbcd366*) and TB (*wPt2740-Xcfa2076*) with a LOD score of 3.4 and 2.2, respectively. However, no significant QTL was detected for RB, which could be attributed to a high CV value (29%) observed for RB.

QTLs for yield component traits SWPS and KPS were detected with LOD scores ranging from 2.4 to 3.5 and PVE ranging from 9.1% to 15.2%. The QTL detected for KPS (*Xwmc664-Xbarc67*) co-localized with that for SWPS. The same marker-interval was associated with KPSM, GY, and SPSM in the earlier studies conducted using the same mapping population, and was designated as region 2 [10]–[12]. Presence of the coincident QTLs in this region was also observed using a ‘mirror population’ of 90 RICLs, which was derived from a cross between WI and one of its substitution lines, WI(CNN3A) [14].

Greenhouse data analysis detected a QTL for TKW that was flanked by markers *Xgwm218-Xbf20313*. Field data analysis also detected *Xgwm218* as one of the associated markers flanking QTLs for TKW, GY and PH [11], [12]. However, in the present field data analysis a QTL for SPSM was found associated with *Xgwm218*. Interestingly, presence of overlapping QTLs identified in the present and the earlier studies was validated by the analysis of the ‘mirror population’, where the QTLs for GVWT, SPSM, and PH were found associated with *Xgwm218* [14]. Similarly a QTL for HD in the marker interval *wPt2698-wPt2740* was detected using greenhouse data, however no QTL for HD was detected in the same marker interval using the field data. Instead, a QTL each for GVWT and SPSM was detected in this marker interval. The LOD score for the HD QTL detected using the greenhouse data was 3.2 and PVE was 18.3%. The presence of *earliness per se* (*Eps*) genes on chromosome 3A was also reported in the earlier studies [9].

In the greenhouse, average plant heights of WI and CNN were significantly different. A reduction in plant height of 17 cm by the introduction of WI chromosome 3A in CNN background with a skewed distribution of plant height towards the WI allele among the RICLs was observed (table S3). The QTL analysis for PH identified a QTL each in the marker intervals *Xpsp3047-XksuA6* and *Xcdo638-Xbarc356*. These QTLs are consistently detected between field and greenhouse data (table 1 and figure 1), and also in the previous studies using the same mapping population [11].

### QTL Analysis Using Field Data

#### (i) Heading Date (HD)

**Figure 2:**
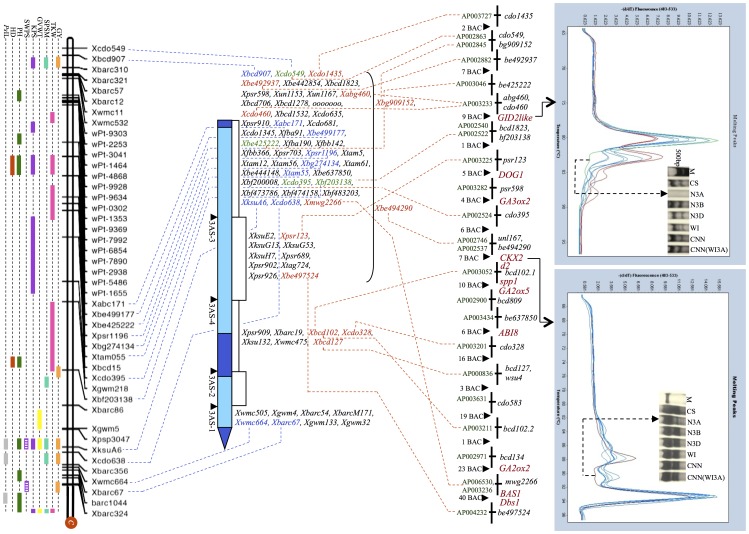
Alignment of cytogenetic-ladder map of wheat chromosome 3AS, showing locations of QTLs for 10 agronomical traits (confidence intervals represented by colored rectangular/square boxes), with rice chromosome 1 to identify candidate genes (CGs; highlighted in red) underlying the QTLs. Genomic locations of two CGs were confirmed via melt curve analysis and PAGE using the genomic DNAs derived from the nulli-tetrasomic lines of wheat group 3 chromosomes, wheat cultivars Chinese Spring (CS), Wichita (WI), Cheyenne (CNN) and its substitution line [CNN(WI3A)]. On the cytogenetic map markers shown in blue are common between genetic and cytogenetic maps, the markers shown in red are common between the rice BAC-contig and cytogenetic map, and markers shown in green are common among all the maps. C = Centromere; GY = grain yield; KPSM = kernels per square meter; TKW = 1000-kernel weight; SPSM = spikes per square meter; GVWT = grain volume weight; KPS = kernels per spike; SWPS = seed weight per spike; PH = plant height; HD = heading date; PsIL = *Pseudocercosporella* induced lodging.

QTL analysis of the field data collected over 13 environments and of data pooled over environments for the years 2000, 2001, 2005 and 2006, allowed identification of four QTLs for HD. Out of these four QTLs, two QTLs (*wPt3041-wPt4868* and *Xbg27413-Xtam055*) were consistently detected over two environments, whereas the remaining QTLs were detected in a single environment each. The LOD scores ranged from 2.4 to 3.2 and PVE ranged from 9.64% to 14.11%. The distribution of phenotypic data for HD was bimodal. In an earlier study, performed using the same mapping population, the phenotypic data recorded on HD was treated as a qualitative trait and assigned to chromosome arm 3AS as *earliness per se* (*Eps*) locus distal to a RFLP marker *Xcdo549* [9]. Presence of an earliness locus on chromosome 3A was also reported in earlier studies [6], [34]–[36]. The two QTLs for HD (marker intervals: *wPt_3041-wPt_4868* and *Xbg27413-Xtam055*) detected in the present study mapped proximal to *Xcdo549*. Although, *Xcdo549* and *Xtam055* (flanking two HD QTLs) are separated by a long genetic distance of 68.6 cM on the linkage map both markers were localized to the same deletion bin, 3AS-3 FL 0.71 (size 102.95 Mb) on the physical map (figure 1 and figure 2). Another marker (*Xbarc45*), from the same deletion bin that contains QTLs for HD and the *Eps* locus, was recently shown to be associated with days to ear emergence [37] and seems to correspond with the HD QTLs detected during the present study. Moreover, mapping of a wheat *FT* paralogue *TaFT2* to chromosome 3A^m^S [38] signifies the importance of this chromosomal region in providing adaptive advantage to wheat.

#### (ii) Plant Height (PH)

In case of PH, 17 QTLs were detected in 19 different environments (including pooled environments for the years 2000, 2001, 2005 and 2006); the LOD scores ranged from 2.3 to 8.1 and PVE ranged from 7.64% to 24.43%. Ten of the 17 QTLs were detected in more than one environment (table 2 and table S4). Two of these QTLs (*Xpsp3047-XksuA6* and *Xcdo638-Xbarc356*) were also detected with the greenhouse data. As expected, WI 3A contributed allele(s) with negative additive effect. Therefore, reduction in PH was observed in RICLs carrying the WI allele(s) compared to that carrying the CNN allele(s). As previously shown, the QTLs influencing plant height were detected on both the long as well as the short arm of chromosome 3A [5], [12]. Markers associated with the PH QTLs detected on 3AS (*XksuA6, Xcdo638* and *Xgwm5*) mapped to the same deletion-bin, 3AS-3 (FL 0.71), and most likely correspond with another QTL identified to be associated with *Xwmc532*, which has previously been placed in the same deletion bin [39]. Similarly, the PH QTL bracketed by markers *Xbarc1021* and *Xpsr1205* on 3AL most likely correspond with the QTL associated with *Xwmc388* [40].

**Table 2:** List of consistent QTLs* detected on chromosome 3A using data recorded over fourteen different environment years.

Trait	QTL interval	cM	LOD	R^2^	Additive effect	Position	Year**
HD	*Xbg27413-Xtam055*	3.1	2.8	14.11	−1.14	67.9	99, 00-Lin
	*wPt_3041-wPt_4868*	1.2	3.2	13.50	1.28	21.4	00-Lin, 05-joint
PH	*Xbarc1021-Xpsr1205*	16.6	4.3	16.09	1.40	346.1	01-Md, 01-Lin, 05-Md, 05-Lin, 01-joint, 05-joint
	*Xpsp3047-XksuA6*	7.1	7.2	24.43	−2.15	157.6	00-Lin, 00-Sd, 01-Sd, 05-Md, 05-Sd, 07-Md, 01-Joint, 00-Joint, 05-Joint
	*Xbarc356-Xwmc664*	1.1	8.0	23.88	−2.13	172.6	01-Sd, 05-Md, 05-Sd, 00-joint, 05-joint
	*Xbarc67-Xbarc324*	2.2	8.1	24.34	−2.15	174.8	00-Md, 05-Sd
	*Xhbg491-Xbcd361*	7.1	3.6	15.42	−1.49	291.0	00-Md, 05-Md, 06-Lin, 05-joint
	*Xtam055-Xbcd15*	10.8	4.2	15.97	−0.87	69.0	00-Sd, 00-joint, 06-joint
	*Xbarc57-Xbarc12*	0.6	5.1	19.18	−0.97	11.3	05-Lin, 00-joint, 06-joint
	*wPt_3041-wPt_4868*	1.2	3.7	13.93	−1.94	21.4	05-Lin, 06-Lin, 05-joint
	*wPt_2938-Xabc171*	15.1	4.0	17.13	−0.89	25.4	05-Lin, 06-Lin, 06-joint
	*wPt_9303-wPt_2253*	1.3	5.0	18.84	−0.95	20.3	06-Lin, 06-joint
TKW	*Xwmc559-Xhbg491*	1.9	4.4	14.35	0.38	284.8	01-Lin, 06-joint
	*wPt_3041-wPt_4868*	1.2	4.4	19.22	−0.39	21.8	01-Md, 00-joint, 01-joint, 05-joint, 06-joint
	*wPt_9928-wPt_1353*	0.6	4.2	16.00	−0.40	23.2	01-Sd, 01-joint
	*Xbarc324-Xwmc489*	1.2	5.3	18.81	−0.88	177.0	05-Md, 05-Sd, 05-joint
	*Xwmc11-Xwmc532*	1.1	5.6	23.69	−0.44	12.8	05-joint, 06-joint
	*Xbcd366-Xbcd927*	4.6	3.4	13.45	−0.87	197.7	05-Md, 05-joint
KPS	*Xbcd907-Xbarc310*	5.0	4.0	15.04	0.69	5.4	01-Sd, 06-joint
	*Xwmc532-wPt_9303*	6.9	4.8	16.68	0.73	13.8	01-Sd, 06-Md
	*wPt_3041-wPt_4868*	1.2	4.6	16.03	0.71	21.7	01-Sd, 06-Md, 06-joint
	*Xwmc428-Xcfa2262*	2.0	3.6	12.24	0.96	187.2	01-Sd, 06-joint
	*Xbarc1060-Xbcd141*	5.9	5.5	25.25	−1.58	232.7	01-Sd, 05-Md, 01-joint
	*Xpsp3047-XksuA6*	7.1	2.6	14.28	0.90	163.9	00-Lin, 00-joint
	*Xbarc324-Xwmc489*	1.2	2.5	10.59	0.55	176.6	05-Md, 05-joint
	*wPt_1353-wPt_1655*	1.2	4.1	17.64	1.27	23.8	06-Md, 06-joint
GY	*Xbcd907-Xbarc310*	5.0	5.7	15.50	0.18	5.3	99, 07-Md
	*Xpsp3047-XksuA6*	7.1	8.1	30.50	0.09	159.8	00-Lin, 01-Lin, 00-joint, 01-joint, 06-joint
	*Xcdo638-Xbarc356*	5.3	9.6	33.98	0.09	169.1	00-Lin, 01-Lin, 01-Md, 00-joint, 01-joint, 06-joint
	*Xwmc664-Xbarc67*	1.1	8.3	29.24	0.08	173.6	01-Md, 00-joint, 01-joint
	*Xbcd15-Xcdo395*	18.0	3.5	19.80	0.12	87.4	00-Md, 00-joint
GVWT	*Xbarc1060-Xbcd141*	5.9	3.1	21.32	0.66	230.6	99, 06-Md
	*Xbcd372-Xhbg227*	15.6	3.9	21.18	0.26	243.0	99, 05-Sd
	*Xpsp3047-XksuA6*	7.1	4.7	17.52	0.33	161.6	01-Lin, 05-Lin, 05-Md, 06-Md, 01-joint, 05-joint, 06-joint
	*Xcfa2183-wPt_2967*	3.6	4.9	24.26	0.27	305.6	00-Lin, 01-Lin, 01-Sd, 00-joint, 01-joint
	*Xcfa2262-Xbcd366*	8.3	5.1	27.60	0.26	193.1	00-Sd, 01-Sd, 06-Lin
	*Xcfa2076-Xwmc388*	6.0	3.3	14.76	−0.38	336.1	01-Sd, 07-Md
	*Xbarc1021-Xpsr1205*	16.6	3.1	18.29	−0.73	340.3	01-Sd, 07-Md
	*Xbcd361-Xgwm155*	7.4	4.8	21.92	0.39	299.8	00-Lin, 06-Md, 07-Md, 05-joint
	*Xbarc324-Xwmc489*	1.2	5.8	23.26	0.23	177.0	00-Sd, 01-joint
	*Xhbg284-Xwmc428*	5.9	6.6	28.53	0.26	183.4	00-Sd, 01-joint
	*Xbarc86-Xgwm5*	9.2	3.6	16.50	0.51	156.0	05-Lin, 06-joint
SPSM	*Xbcd907-Xbarc310*	5.0	2.6	15.15	20.51	7.5	99, 00-Md, 00-joint
	*Xpsp3047-XksuA6*	7.1	6.4	26.03	20.03	159.8	01-Lin, 01-joint, 06-joint
	*Xcdo638-Xbarc356*	5.3	4.1	17.40	20.67	171.4	01-Lin, 01-joint
	*Xbarc1021-Xpsr1205*	16.6	3.6	17.74	14.72	344.9	01-Lin, 06-Lin, 01-joint
	*Xbcd1555-Xbcd22*	1.7	2.1	8.21	14.35	236.3	01-Sd, 01-joint, 05-joint
	*Xbarc324-Xwmc489*	1.2	5.1	19.95	26.70	177.0	00-Lin, 06-Lin, 06-joint
	*Xcdo395-Xgwm218*	8.2	2.7	12.47	25.18	99.7	05-Md, 06-Md
	*Xcfa2183-wPt_2698*	3.6	3.4	12.69	21.09	304.0	05-Md, 06-Md
	*Xhbg284-Xwmc428*	5.9	6.2	25.67	21.55	185.3	06-Linl 06-joint
	*Xhbg227-wPt_1562*	19.0	2.5	21.39	−30.81	268.1	06-Lin, 07-Md

** For years 2005 and 2008 data was recorded at Lincoln and Pullman, respectively; SPSM = spike/square meter, PH = plant height, HD = heading date, TKW = 1000-kernel weight, GY = grain yield, KPSM = kernels/square meter, GVWT = grain volume weight, and KPS = kernels per spike.

* For the purpose of identifying consistent QTLs, the QTLs detected in overlapping marker intervals with one common marker flanking the region were treated as the same QTL. Among these QTLs the marker interval showing the highest LOD score and R^2^ value was documented in the table (cf. table S4 for complete list of QTLs).

#### (iii) Grain Yield (GY)

Eleven QTLs were detected in 16 different environments including pooled data for the years 2000, 2001, 2005 and 2006. The LOD scores of the QTLs ranged from 2.5 to 9.6 and PVE ranged from 9.36% to 33.98% (table 1, figure 1 and figure 2). Five of these QTLs were consistently detected in two to six of the studied environments. All QTLs showed positive additive effects with WI alleles favoring higher grain yield. In earlier studies conducted using the same mapping population, QTLs for grain yield were localized to overlapping marker intervals (*Xbarc86-Xwmc388.1*, *Xcdo638-Xbarc67*, *Xmc664-Xbarc67* and *Xstm6352-Xwmc428*) [10]–[12]. In the present study, the yield QTLs mapped to the same regions but to narrower marker intervals (figure S1). These results clearly showed that major QTL for grain yield exist both in the telomeric and subtelomeric regions of 3AS. Compared to earlier studies using the same mapping population, we detected a higher number of grain yield QTLs. This result can be attributed to the increase in marker density in the present genetic map, which might have resulted in partitioning of the effect to the adjacent marker intervals leading to identification of many more QTLs in consecutive marker intervals with lower LOD scores and PVE values.

#### (iv) Grain Volume Weight (GVWT)

The grain volume weight or test weight is an important trait as it is associated with the flour yield during wheat milling. Across all environments, the substitution line CNN(WI3A) showed a higher GVWT compared to CNN, however the variation was statistically non-significant. Fifteen QTLs were detected for GVWT in 15 of the 17 environments (including pooled data for the years 2000, 2001, 2005 and 2006). LOD scores for the QTLs ranged from 2.3 to 6.6 and PVE ranged from 7.05% to 28.53% (table 2, figure 1 and figure 2). Out of the 15 QTLs, 11 were detected in two or more environments (table S4). Twelve of the 15 QTLs showed positive additive effects and the remaining showed negative additive effects suggesting that the two parents have complimentary genes/alleles for GVWT. All four major chromosomal regions associated with the GVWT using an extended set of 223 RICLs were also identified in the present study along with the detection of some additional QTLs [12] (figure S1). These results highlight the value of increasing marker density in QTL analysis. The previous study, although conducted on a larger population, had 32 markers compared to 81 in the present study. The GVWT QTL in marker interval *Xbarc19*-*Xcfa2262* coincided with the previously detected meta-QTLs one each for grain width and grain length/width localized to the marker interval *Xcfa2262-Xbcd366* [1].

#### (v) 1000-Kernel Weight (TKW)

Altogether 11 QTLs were detected for TKW in 10 of the 16 environments (including pooled data for the years 2000, 2001, 2005 and 2006) with the LOD scores ranging from 2.3 to 5.6 and PVE from 9.31% to 26.82%. Six of these QTLs were consistent over environments. Seven QTLs showed negative additive effects and four showed positive additive effects. A majority of the QTLs carried favorable alleles from CNN, whereas the favorable alleles for the remaining QTLs were contributed by WI. These results agreed with the earlier findings for TKW [8]–[10]. The consistent QTLs for TKW were detected on both arms of chromosome 3A. QTLs for TKW in two chromosomal regions, *Xbcd361-Xgwm155* and *wPt-3041-wPt-4868* were also detected in an earlier study [41]. The chromosomal region bracketed by markers *Xcfa219* and *wPt-2698* that was reported to contain QTL for grain length [1], coincided with the chromosome region flanked by *Xhbg491* and *wPt-2698* harboring QTLs for TKW, GVWT and SPSM in the present study (figure 1). A few minor QTLs for TKW were also detected in the present study that escaped detection in the previous studies using the same mapping population probably due to lack of resolution (table S4).

#### (vi) Spikes/Square Meter (SPSM)

As many as 14 QTLs were detected for SPSM in 14 of the 16 studied environments (including pooled environments for the years 2000, 2001, 2005 and 2006). The LOD scores of these QTLs ranged from 2.1 to 7.6 and PVE from 30.81% to 32.31% (table 2, figures 1 and 2). For 12 of these QTLs WI contributed the favorable alleles whereas for the remaining two QTLs the favorable alleles were contributed by CNN. Ten QTLs were consistently detected across environments and of these two were previously detected (figure S1) [8]–[10]. Additional QTLs detected during the present study escaped detection in the earlier studies probably due to a lower marker density. As SPSM positively contributes to GY and is mainly determined by number of tillers per plant, expectedly QTLs for tiller number were previously reported in overlapping marker intervals [42], [43].

#### (vii) Kernels/Spikes (KPS)

A total of 11 QTLs were detected for KPS from 10 of the 16 studied environments. Eight of these 11 QTLs were consistently detected over two or more environments. The LOD scores of the QTLs ranged from 2.4 to 5.5 and the PVE ranged from 9.06% to 25.25%. For eight of these QTLs the WI alleles showed positive additive effects, whereas the remaining showed negative effects. As observed for other traits, all previously reported QTLs for KPS were localized to narrower intervals along with the detection of additional QTLs (figure S1). These observations suggested that increasing marker density on the genetic linkage map not only increased resolution but also increased detection power, as minor QTLs that escaped detection in the previous studies were detected in the present study.

#### (viii) *Pseudocercosporella* Induced Lodging (PsIL)

Five QTLs were detected for *Ps*IL using data from a single environment. The LOD scores ranged from 2.7 to 3.4 and PVE ranged from 12.61% to 15.46% (table 2, figure 1 and figure 2). All QTLs showed negative additive effects with CNN alleles showing susceptibility to *Ps*IL. Perhaps coincidently, QTLs for the resistance against *Pseudocercosporella* induced strawbreaker foot-rot localized to the same marker interval where the PH QTLs mapped (*Xpsp3047*, *XksuA6* and *Xcdo638*). Associations between plant height and resistance to *Fusarium* head blight caused by *F. graminearum*, powdery mildew caused by *Blumeria graminis* and eyespot caused by *Oculimacula acuformis* and *O. yallundae* were reported in wheat and barley [44]. The comparative analysis of the regions harboring *Ps*IL QTLs detected in this study with corresponding regions on wheat chromosomes 3B and rice chromosome 1 also showed overlapping distribution of genes conferring resistance against a number of fungal diseases and genes regulating plant height (figures 1, S2 and S3). As alleles for both disease susceptibility and increased plant height are contributed by CNN, it is likely to see a positive correlation between the two traits, where selection for reduced plant height is expected to confer resistance against *Pseudocercosporella* induced lodging.

### Comparison of QTL Analyses Conducted on Field and Greenhouse Data

An objective of this study was to compare quantitative trait data collected in the field with that collected in a greenhouse especially to reliably score traits with low heritability. Our results suggested that the greenhouse data showed less variability in comparison with the field data and also lead to the identification of significantly fewer QTLs in comparison with the field data. For instance, the QTL analysis for four common traits (HD, PH, KPS and TKW) using greenhouse data resulted in the identification of a total of five QTLs, one each for HD, KPS and TKW, and two QTLs for PH. Whereas 52 QTLs were detected using field data collected over multiple environment (including pooled environments). A major reason for identifying fewer QTLs in the greenhouse compared to the field data is due to minimization of genotype × environment and QTL × environment interactions, which have been earlier documented for chromosome 3A using the field data recorded on the same mapping population [2], [10], [45]. A less likely explanation for the large number of QTLs detected using the multi-environment field data is due to identification of false positives at ≤5% error rate, which can be easily avoided by looking only at the consistent QTLs.

For HD a unique negative effect QTL (*wPt_2698-wPt_2740*) with a LOD score of 3.2 and PVE of 18.36% was detected from the greenhouse data analysis. Similarly a unique QTL each for TKW and KPS respectively in the marker intervals *Xgwm218-Xbf20313* and *Xwmc664-Xbarc67* was detected from the greenhouse data analysis. Both of these QTLs have negative additive effects, and LOD scores and PVE values comparable to the QTLs detected from the field data analysis. These QTLs however were not detected in the field data analysis performed in the present study, but were consistently detected in the earlier studies performed on the same population or other populations (see above). These results could be explained by the fact that increase in marker density on the genetic linkage map may allow partitioning of the effect to the adjoining marker intervals often making it un-detectable under the defined parameters. In addition, the two QTLs detected for PH were both consistently detected in field as well as greenhouse data analyses. The PVE values and the LOD scores of the QTLs detected from the greenhouse data were comparable to the mean of LOD scores and PVE values for the similar QTLs identified from multi-environment field data analysis. Thus, it is apparent from the present analysis that the greenhouse data can complement the field data to study low heritability traits, and is a valuable resource to reliably dissect quantitative traits with limited investment of time and money.

### Comparative Mapping and Identification of Candidate Genes

To demarcate rice genomic regions corresponding with the QTLs for GY and other agronomic traits present on wheat chromosome arm 3AS, DNA sequences of 36 (51.43%) of the 70 DNA markers were compared with the rice genomic DNA sequences using various ‘BLAST’ functions. The remaining 34 DNA markers were not used for the analysis as their sequences were not publicly available (table S1). Of the 36 sequences, 20 (55.56%) detected a high level of sequence homology (e-value ≤10^−20^ to 0.0) with the rice genomic DNA sequences (figure 2 and table S1). The remaining 16 (44.44%) sequences, however, did not identify any ortholog in the rice genome. Sequences corresponding to 3 (15%) of the 20 sequences were present in more than one copy in the rice genome (table S1). The wheat chromosome arm 3AS showed maximum homology to rice chromosome 1, as 16 of the 20 (80%) sequences identified their orthologs on rice chromosome 1 (table S1). Homologues for the remaining 4 of the 20 wheat sequences were present on 4 other rice chromosomes (table S1). Distribution of the 16 wheat sequences on rice chromosome 1 is shown in figure 2. The collinear region identified on the rice chromosome 1 spans all major QTLs for yield and other agronomic traits, identified on 3AS.

It is generally difficult to align QTL harboring regions across genera, but due to relatively extensive genome conservation among cereals, it is possible to superimpose QTLs identified among genus. To achieve this objective, the rice genomic DNA sequence was used as surrogate where flanking markers of rice and wheat QTLs were placed to determine correspondence among QTLs for related traits. In some instances, due to lack of sequence information, other associated markers selected on the basis of present/previous mapping information and/or markers mapping to the same deletion bin were used for this purpose. For instance, markers associated with the two HD QTLs identified on 3AS in the present study showed correspondence with a region on rice chromosome 1 carrying a flowering time QTL, *dth1.1* (figure S2) [46]. Similarly, the evolutionarily conserved regions influencing grain yield on short arm of rice chromosome 1 detected in a number of studies [3] corresponded well with the wheat grain yield QTLs, giving new insight into the domestication history of the cereals. A chromosomal region flanked by *Xhbg491* and *wPt-2698*, harboring QTLs for TKW, GVWT and SPSM in the present study corresponded with the region harboring QTLs for grain weight, tiller number and grain yield in proximity of the Lax1 gene in rice (figure S2) [3]. Similarly, QTLs for tillers per plant detected on both long and short arms of rice chromosome 1 corresponded with the QTLs for SPSM (in wheat spikes/m2 represents tillers/m2) in the present study (figure 1 and figure 2) [3]. Co-localizing QTLs for primary, secondary branch number, and panicle weight in rice were reported in chromosome region, which corresponds with the region (*wPt-3041-wPt-1464*), where QTL for KPS was detected in the present study [47]. In general, our data suggested high correspondence in the QTL harboring regions in rice and wheat, which might be a consequence of selection for similar traits during the domestication process of different cereals [48].

With the objective to identify candidate genes (CGs) and to increase marker density in the regions underlying the QTLs of interest, we studied the gene-content of rice BAC/PAC clones spanning the regions underlying QTLs, annotated each gene and compared identified genes with the wheat ESTs. We also placed 94 yield-contributing genes so far cloned from cereals on the rice genomic DNA sequence (data shown for only rice chromosome 1 in figure S2) and determined the location of those sequences that underlie yield QTLs on 3AS (figure 2). Genomic locations of two of these candidate genes (*CKX2* and *GID2-like*) were also confirmed by HRM melt curve analysis and PAGE using gene-specific STS (sequence tagged site) primers [49] on wheat aneuploid stocks (figure 2). To align the region harboring QTL(s) for grain yield with the BAC-contig of 3B and to increase the marker density in this region, the rice BAC/PAC sequences spanning this region were also compared against BAC end sequences derived from chromosome 3B and 3AS specific BAC libraries. The analysis allowed identification of BAC-end sequences that align with the corresponding region of BAC scaffold assembled for chromosome 3B (figure S3). The identified BAC-end sequences were used for the development of STS or sequence tagged microsatellite site (STMS) markers that are currently being used for the development of high-resolution maps of the specific regions (data not shown).

### Deletion Based Physical Mapping

Since recombination is highly uneven on wheat chromosomes [13], it is very important to know the physical location of genes/QTLs before any further characterization is done. Keeping this in mind, the physical location of QTLs was determined by comparing the deletion line-based physical map with the integrated genetic linkage map of chromosome 3A (figure 1 and figure 2). Comparison of genetic and physical maps has shown detailed distribution of recombination on wheat chromosomes and has shown hot and cold spots of recombination along the length of chromosome 3A [13]. The QTLs for grain yield present in the marker intervals *Xcdo549-Xbarc310* and *Xpsp3047-Xbarc356* reside in the high-recombination regions. In comparison, the region containing QTLs for SWPS, KPS, *Ps*IL and SPSM, which are flanked by markers *Xwmc664* and *Xwmc489,* has a low level of recombination. This makes the *Xwmc664* and *Xwmc489* QTL interval less amenable to map-based cloning (figure 1).

To increase the utility of the current genetic linkage map, we aligned the current map with other available genetic maps of chromosome 3A, and assembled a composite genetic map using the CMap function (figure S1). This composite map when aligned with other genetic maps for chromosome 3A, allowed placement of QTLs for a variety of agronomic and quality traits mapped in other mapping populations, on our map. Specifically QTLs for dough score (Dsc), bread score (Bsc), dough strength (W), bread loaf volume (Bvol), grain protein content (GPC), *Fusarium* head blight (FHB) resistance, grain color and preharvest sprouting (PHS) tolerance were placed. This analysis gave a broader view of genes mapping to chromosome 3A.

### Perspective

The present study describes comprehensive genetic and physical maps of wheat chromosome 3A and their use in demarcating chromosomal regions harboring genes/QTLs influencing agronomically important traits. Comparison of a high-density genetic linkage map with the integrated physical map of chromosome 3A led to identification of chromosomal regions carrying cluster of coinciding QTLs for various agronomically important traits. Rice-wheat synteny allowed identification of candidate genes (CGs) underlying some of the QTL-clusters. A similar comparison with 3AS [50] and 3BS [51] BAC-end sequences lead to the identification of corresponding region(s) on the BAC-based physical map of chromosome 3B [52]. This activity will not only allow us to develop new markers for the region of interest, but will also allow us to anchor genetic map with the BAC-based physical maps of chromosome 3A and 3B.

## Supporting Information

Figure S1 **Alignment of chromosome 3A genetic linkage maps and comparative mapping of quality trait loci.** A. Consensus map including 191 markers, B. map prepared using 95 CNN(WI3A) RICLs and 20 markers [10], C. map prepared using 223 CNN(WI3A) RICLs and 41 markers, D. map prepared using 95 CNN(WI3A) RICLs and 81 markers, and E. Five major regions previously identified to harbor QTLs for yield and component traits. GY = grain yield, SPSM = spike/square meter, KPS = kernels/spike, TKW = 1000-kernel weight, PHT = plant height, and HD = heading date. GPC = grain protein content, Dsc = dough score, Bsc = bread score, W = dough strength, Bvol = bread loaf volume, FHB = *Fusarium* head blight resistance, GC = grain colour, and PHS = preharvest sprouting tolerance.
(PDF)

Figure S2 **Rice chromosome 1 showing the genomic locations of different yield contributing genes.** On left the short arm of rice chromosome 1 is magnified to show the map locations of two flowering time QTLs (*dth1.1a* and *dth1.1b*), and candidate genes underlying these QTLs.
(PDF)

Figure S3 **A magnified view of the Region 1 flanked by markers** ***Xcdo549*** **and** ***XBE425222*** **(CJ582163), harboring QTLs for grain yield, kernels/squire meter, spikes/squire meter and kernels/spike** (figure S1) [11]. Rice genomic DNA sequences corresponding with the wheat region were used as surrogate to BLAST against ESTs mapped to wheat group 3 chromosomes, unmapped ESTs and wheat chromosome 3A and 3B BAC end sequences. On extreme right the BAC-contigs of wheat chromosome 3B spanning the regions of interest is shown. The ESTs (mapped and unmapped) and BAC end sequences (shown in blue) identified in the study were used to design new markers for high-resolution mapping of the QTLs identified in this region.
(PDF)

Table S1 **List of molecular markers used for comparative sequence analysis with rice genomic DNA sequences.**
(DOCX)

Table S2 **Correlations observed for eight agronomical traits recorded in greenhouse on the 95 RICLs 3A-mapping population.**
(DOCX)

Table S3 **Parental values, range and heritability of eight agronomical traits recorded in greenhouse on WI, CNN, CNN(WI3A) and 95 RICLs.**
(DOCX)

Table S4 **List of QTLs detected on chromosome 3A in different environment-years for seven agronomical traits using 95-RICL 3A mapping population.**
(DOCX)
