# Supplementary material for: Genetic Dissection of Yield and Its Component Traits Using High-Density Composite Map of Wheat Chromosome 3A: Bridging Gaps between QTLs and Underlying Genes
Source: PLoS One. 2013 Jul 24;8(7):e70526. doi: 10.1371/journal.pone.0070526 (PMC3722237; doi:10.1371/journal.pone.0070526)
Supplement: Figure S1 — Alignment of chromosome 3A genetic linkage maps and comparative mapping of quality trait loci. A. Consensus map including 191 markers, B. map prepared using 95 CNN(WI3A) RICLs and 20 markers [10], C. map prepared using 223 CNN(WI3A) RICLs and 41 markers, D. map prepared using 95 CNN(WI3A) RICLs and 81 markers, and E. Five major regions previously identified to harbor QTLs for yield and component traits. GY = grain yield, SPSM = spike/square meter, KPS = kernels/spike, TKW = 1000-kernel weight, PHT = plant height, and HD = heading date. GPC = grain protein content, Dsc = dough score, Bsc = bread score, W = dough strength, Bvol = bread loaf volume, FHB = Fusarium head blight resistance, GC = grain colour, and PHS = preharvest sprouting tolerance. (PDF) [file pone.0070526.s001.pdf]

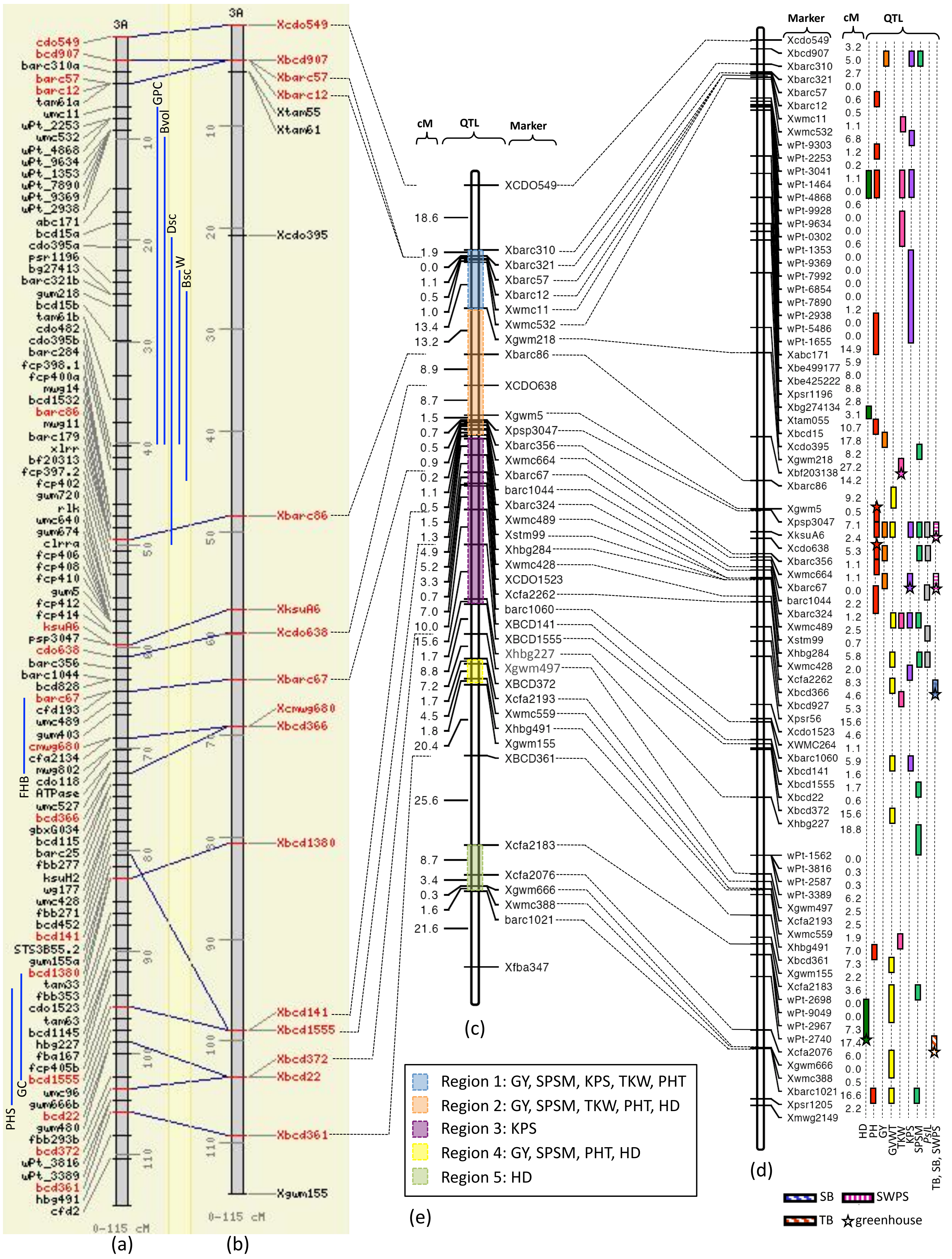
3A
cdo549
bcd907
barc310a
barc57
barc12
tam61a
wmc11
wPt_2253
wmc532
wPt_4868
wPt_9634
wPt_1353
wPt_7890
wPt_9369
wPt_2938
abc171
bcd15a
cdo395a
psr1196
bg27413
barc321b
gwm218
bcd15b
tam61b
cdo482
cdo395b
barc284
fcp398.1
fcp400a
mwg14
bcd1532
barc86
mwg11
barc179
xlrr
bf20313
fcp397.2
fcp402
gwm720
rlk
wmc640
gwm674
clrra
fcp406
fcp408
fcp410
gwm5
fcp412
fcp414
ksuA6
psp3047
cdo638
barc356
barc1044
bcd828
barc67
cfd193
wmc489
gwm403
cmwg680
cfa2134
mwg802
cdo118
ATPase
wmc527
bcd366
gbxG034
bcd115
barc25
fbb277
ksuH2
wg177
wmc428
fbb271
bcd452
bcd141
STS3B55.2
gwm155a
bcd1380
tam33
fbb353
cdo1523
tam63
bcd1145
hbg227
fba167
fcp405b
bcd1555
wmc96
gwm666b
bcd22
gwm480
fbb293b
bcd372
wPt_3816
wPt_3389
bcd361
hbg491
cfd2
0-115 cM
GPC
Bvol
Dsc
W
Bsc
FHB
GC
PHS
(a)
3A
Xcdo549
Xbcd907
Xbarc57
Xbarc12
Xtam55
Xtam61
Xcdo395
Xbarc86
XksuA6
Xcdo638
Xbarc67
Xcmwg680
Xbcd366
Xbcd1380
Xbcd141
Xbcd1555
Xbcd372
Xbcd22
Xbcd361
Xgwm155
0-115 cM
(b)
cM
QTL
Marker
XCDO549
18.6
1.9
0.0
1.1
0.5
1.0
13.4
13.2
8.9
8.7
1.5
0.7
0.5
0.9
0.2
1.1
0.5
1.5
1.3
4.9
5.2
3.3
0.7
7.0
10.0
15.6
1.7
8.8
7.2
1.7
4.5
1.8
20.4
25.6
8.7
3.4
0.3
1.6
21.6
Xbarc310
Xbarc321
Xbarc57
Xbarc12
Xwmc11
Xwmc532
Xgwm218
Xbarc86
XCDO638
Xgwm5
Xpsp3047
Xbarc356
Xwmc664
Xbarc67
barc1044
Xbarc324
Xwmc489
Xstm99
Xhbg284
Xwmc428
XCDO1523
Xcfa2262
barc1060
XBCD141
XBCD1555
Xhbg227
Xgwm497
XBCD372
Xcfa2193
Xwmc559
Xhbg491
Xgwm155
XBCD361
Xcfa2183
Xcfa2076
Xgwm666
Xwmc388
barc1021
Xfba347
(c)
Region 1: GY, SPSM, KPS, TKW, PHT
Region 2: GY, SPSM, TKW, PHT, HD
Region 3: KPS
Region 4: GY, SPSM, PHT, HD
Region 5: HD
(e)
Marker
cM
QTL
Xcdo549
Xbcd907
Xbarc310
Xbarc321
Xbarc57
Xbarc12
Xwmc11
Xwmc532
wPt-9303
wPt-2253
wPt-3041
wPt-1464
wPt-4868
wPt-9928
wPt-9634
wPt-0302
wPt-1353
wPt-9369
wPt-7992
wPt-6854
wPt-7890
wPt-2938
wPt-5486
wPt-1655
Xabc171
Xbe499177
Xbe425222
Xpsr1196
Xbg274134
Xtam055
Xbcd15
Xcdo395
Xgwm218
Xbf203138
Xbarc86
Xgwm5
Xpsp3047
XksuA6
Xcdo638
Xbarc356
Xwmc664
Xbarc67
barc1044
Xbarc324
Xwmc489
Xstm99
Xhbg284
Xwmc428
Xcfa2262
Xbcd366
Xbcd927
Xpsr56
Xcdo1523
XWMC264
Xbarc1060
Xbcd141
Xbcd1555
Xbcd22
Xbcd372
Xhbg227
wPt-1562
wPt-3816
wPt-2587
wPt-3389
Xgwm497
Xcfa2193
Xwmc559
Xhbg491
Xbcd361
Xgwm155
Xcfa2183
wPt-2698
wPt-9049
wPt-2967
wPt-2740
Xcfa2076
Xgwm666
Xwmc388
Xbarc1021
Xpsr1205
Xmwg2149
3.2
5.0
2.7
0.0
0.6
0.5
1.1
6.8
1.2
0.2
1.1
0.0
0.6
0.0
0.0
0.6
0.0
0.0
0.0
0.0
1.2
0.0
0.0
14.9
5.9
8.0
8.8
2.8
3.1
10.7
17.8
8.2
27.2
14.2
9.2
0.5
7.1
2.4
5.3
1.1
1.1
0.0
2.2
1.2
2.5
0.7
5.8
2.0
8.3
4.6
5.3
15.6
4.6
1.1
5.9
1.6
1.7
0.6
15.6
18.8
0.0
0.3
0.3
6.2
2.5
2.5
1.9
7.0
7.3
2.2
3.6
0.0
0.0
7.3
17.4
6.0
0.0
0.5
16.6
2.2
HD
PH
GY
GVWT
TKW
KPS
SPSM
PsL
TB, SB, SWPS
(d)
SB
SWPS
TB
greenhouse
