# Supplementary figures and images for: Genetic Dissection of Yield and Its Component Traits Using High-Density Composite Map of Wheat Chromosome 3A: Bridging Gaps between QTLs and Underlying Genes
Source: PLoS One. 2013 Jul 24;8(7):e70526. doi: 10.1371/journal.pone.0070526 (PMC3722237; doi:10.1371/journal.pone.0070526)

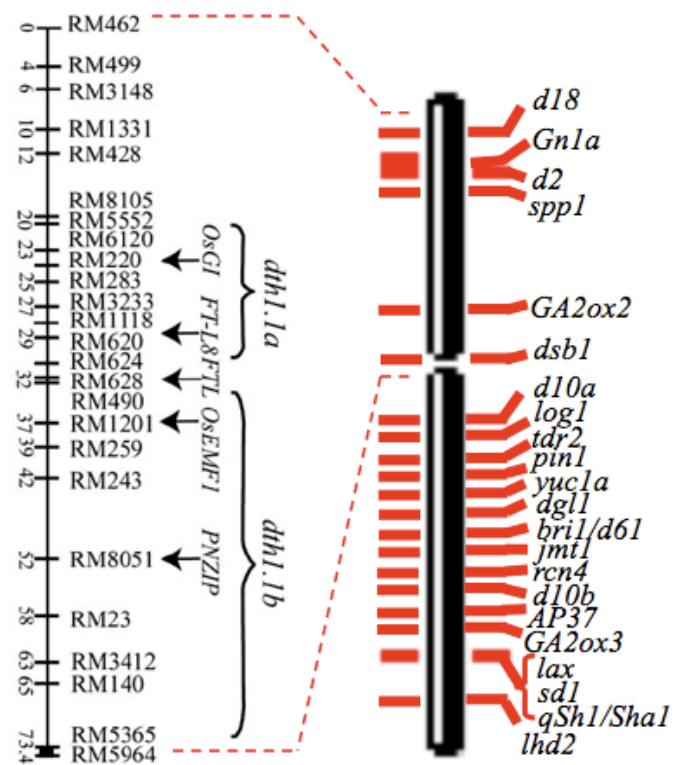

**R1**  
(Gp. 3)

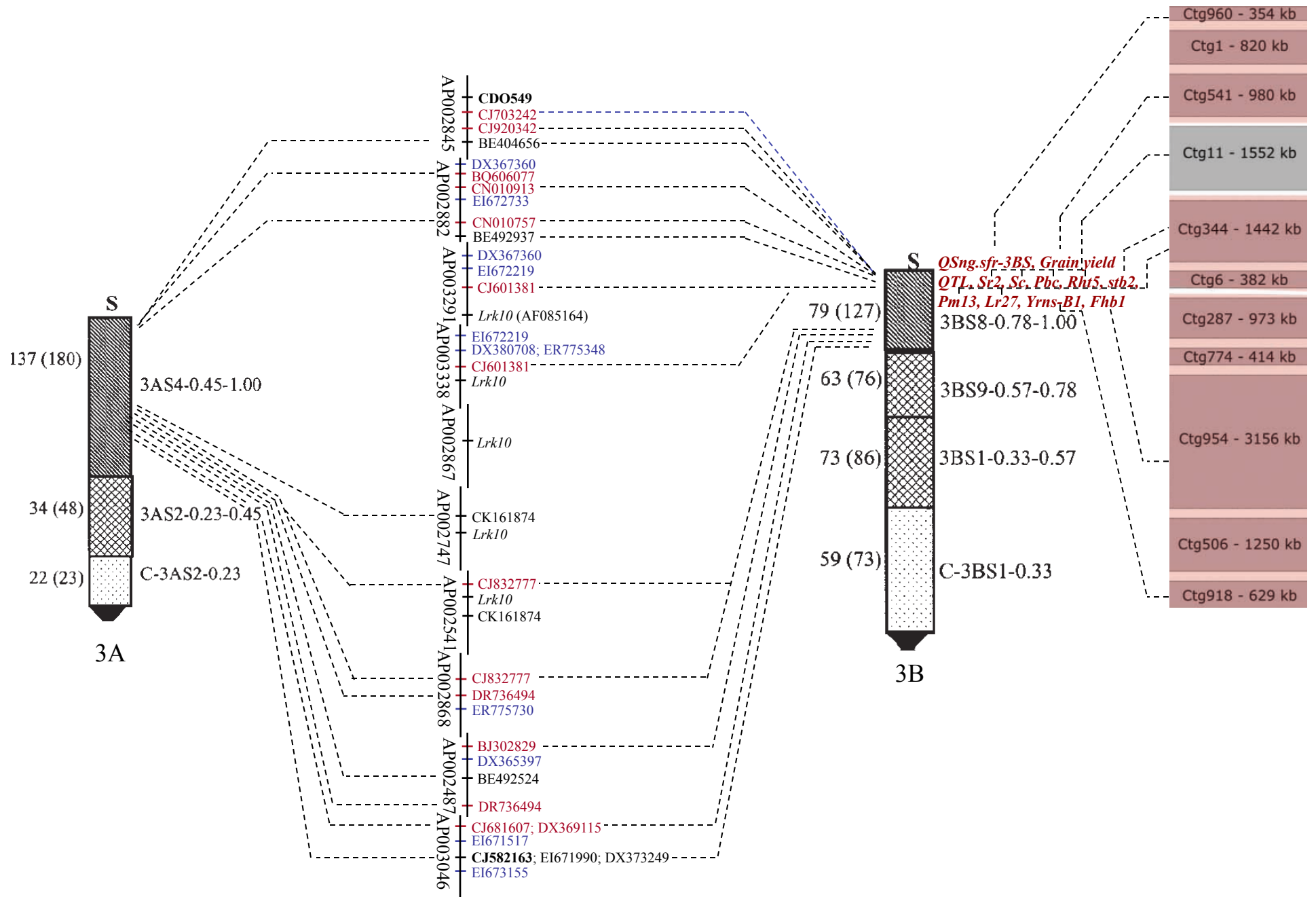

Rice BAC scaffold

Supplement: Figure S2 — Rice chromosome 1 showing the genomic locations of different yield contributing genes. On left the short arm of rice chromosome 1 is magnified to show the map locations of two flowering time QTLs (dth1.1a and dth1.1b), and candidate genes underlying these QTLs. (PDF) [file pone.0070526.s002.pdf]

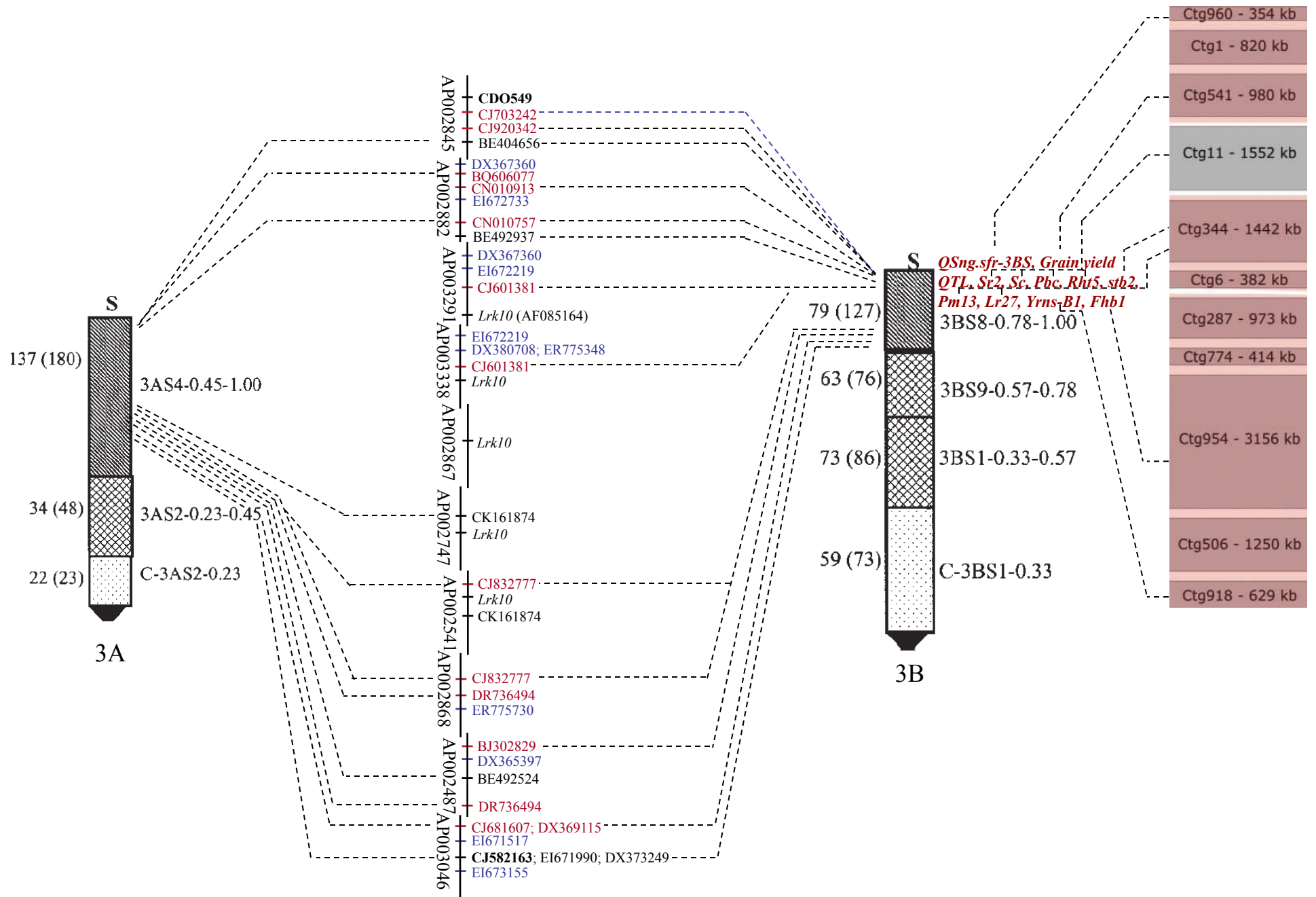

Rice BAC scaffold

Supplement: Figure S3 — A magnified view of the Region 1 flanked by markers Xcdo549 and XBE425222 (CJ582163), harboring QTLs for grain yield, kernels/squire meter, spikes/squire meter and kernels/spike (figure S1) [11]. Rice genomic DNA sequences corresponding with the wheat region were used as surrogate to BLAST against ESTs mapped to wheat group 3 chromosomes, unmapped ESTs and wheat chromosome 3A and 3B BAC end sequences. On extreme right the BAC-contigs of wheat chromosome 3B spanning the regions of interest is shown. The ESTs (mapped and unmapped) and BAC end sequences (shown in blue) identified in the study were used to design new markers for high-resolution mapping of the QTLs identified in this region. (PDF) [file pone.0070526.s003.pdf]
