# Supplementary material for: Genetic Dissection of Yield and Its Component Traits Using High-Density Composite Map of Wheat Chromosome 3A: Bridging Gaps between QTLs and Underlying Genes
Source: PLoS One. 2013 Jul 24;8(7):e70526. doi: 10.1371/journal.pone.0070526 (PMC3722237; doi:10.1371/journal.pone.0070526)
Supplement: Table S1 — List of molecular markers used for comparative sequence analysis with rice genomic DNA sequences. (DOCX) [file pone.0070526.s004.docx]

**Table S1** List of molecular markers used for comparative sequence analysis with rice genomic DNA.

|  |  |  |  |
| --- | --- | --- | --- |
| **Marker** | **Sequence** | **Chromosome** | **BAC** |
| *bcd907* | no | - | - |
| *cdo549/MWG865-5* | yes | 1 | AP002863, AP002845 |
| *cdo1435* | yes | 1 | AP003727 |
| *BE492937* | yes | 1 | AP002882 |
| *BE442854* | yes | - | - |
| *bcd1823* | yes | 1 | AP002522 |
| *psr598* | yes | 1 | AP003339 |
| *unl153* | no | - | - |
| *unl167* | yes | 1 | AP002746, AP002537 |
| *abg460* | yes | 1 | AP003233 |
| *bcd706* | yes | - | - |
| *bcd1278* | yes | - | - |
| *cdo460* | yes | - | - |
| *bcd1532* | yes | - | - |
| *cdo635* | no | - | - |
| *psr910* | no | - | - |
| *abc171* | no | - | - |
| *cdo681* | no | - | - |
| *cdo1345* | no | - | - |
| *fba91* | no | - | - |
| *BE499177* | yes | - | - |
| *BE425222* | yes | 1 | AP003046 |
| *fba190* | no | - | - |
| *fbb142* | no | - | - |
| *fbb366* | no | - | - |
| *psr703* | no | - | - |
| *psr1196* | yes | - | - |
| *tam5* | no | - | - |
| *tam12* | no | - | - |
| *tam56* | no | - | - |
| *BG274134* | yes | 8 | - |
| *tam61* | no | - | - |
| *BE444148* | yes | - | - |
| *BE637850* | yes | 1 | AP003434 |
| *BF200008* | no | - | - |
| *cdo395* | yes | 2 | - |
| *BF203138* | yes | - | - |
| *BF473786* | yes | 11 | - |
| *BF474158* | yes | 7 | - |
| *BF483203* | yes | - | - |
| *ksuA6* | no | - | - |
| *cdo638* | yes | - | - |
| *mwg2266* | yes | 1 | AP006530 |
| *KsuE2* | no | - | - |
| *Psr123* | yes | 1 | AP003225 |
| *KsuG13* | no | - | - |
| *KsuG53* | no | - | - |
| *KsuH7* | no | - | - |
| *psr689* | no | - | - |
| *psr902* | yes | - | - |
| *tag724* | no | - | - |
| *psr926* | no | - | - |
| *BE497524* | yes | - | - |
| *psr909* | yes | - | - |
| *barc19* | no | - | - |
| *bcd102* | yes | 1 | AP003052, AP003211 |
| *cdo328* | yes | 1 | AP003201 |
| *Ksu132* | no | - | - |
| *wmc475* | yes | - | - |
| *bcd127* | yes | 1 | AP000836 |
| *wmc505* | yes | - | - |
| *gwm4* | no | - | - |
| *barc54* | no | - | - |
| *barcM171* | no | - | - |
| *wmc664* | no | - | - |
| *barc67* | no | - | - |
| *gwm133* | no | - | - |
| *bg909152* | yes | 1 | AP002845 |
| *BE494290* | yes | 1 | AP002537 |
| *gwm32* | no | - | - |
