# Supplementary material for: Genetic Dissection of Yield and Its Component Traits Using High-Density Composite Map of Wheat Chromosome 3A: Bridging Gaps between QTLs and Underlying Genes
Source: PLoS One. 2013 Jul 24;8(7):e70526. doi: 10.1371/journal.pone.0070526 (PMC3722237; doi:10.1371/journal.pone.0070526)
Supplement: Table S2 — Correlations observed for eight agronomical traits recorded in greenhouse on the 95 RICLs 3A-mapping population. (DOCX) [file pone.0070526.s005.docx]

**Table S2** Correlations observed for eight agronomical traits recorded in greenhouse on the 95 RICLs 3A-mapping population.

|  |  |  |  |  |  |  |  |  |
| --- | --- | --- | --- | --- | --- | --- | --- | --- |
| **Trait** | **HD** | **PH** | **SB** | **RB** | **TB** | **TKW** | **SWPS** | **KPS** |
| HD | 1 |  |  |  |  |  |  |  |
| PH | 0.30^**^ | 1 |  |  |  |  |  |  |
| SB | 0.43^**^ | 0.66^**^ | 1 |  |  |  |  |  |
| RB | 0.28^**^ | 0.45^**^ | 0.62^**^ | 1 |  |  |  |  |
| TB | 0.13 | 0.57^**^ | 0.85^**^ | **0.79^**^** | 1 |  |  |  |
| TKW | -0.22^*^ | 0.08 | 0.12 | **0.37^**^** | 0.33^**^ | 1 |  |  |
| SWPS | -0.22^*^ | **0.61^**^** | **0.53^**^** | **0.40^**^** | **0.59^**^** | 0.54^**^ | 1 |  |
| KPS | -0.23^*^ | **0.66^**^** | **0.55^**^** | **0.35^**^** | **0.58^**^** | 0.37^**^ | 0.96^**^ | 1 |

Bold Face correlation coefficients are 2^nd^ order polynomials, *significant at 95% probability; ** significant at 99% probability

HD = heading date, PH = plant height, SB = shoot biomass, RB = root biomass, TB = total biomass, TKW = 1000-kernel weight, SWPS = seed weight per spike, KPS = kernels per spike
