# Supplementary material for: Genetic Dissection of Yield and Its Component Traits Using High-Density Composite Map of Wheat Chromosome 3A: Bridging Gaps between QTLs and Underlying Genes
Source: PLoS One. 2013 Jul 24;8(7):e70526. doi: 10.1371/journal.pone.0070526 (PMC3722237; doi:10.1371/journal.pone.0070526)
Supplement: Table S3 — Parental values, range and heritability of eight agronomical traits recorded in greenhouse on WI, CNN, CNN(WI3A) and 95 RICLs. (DOCX) [file pone.0070526.s006.docx]

**Table S3** Parental values, range and heritability of eight agronomical traits recoded in greenhouse on WI, CNN, CNN(WI3A) and 95 RICLs.

|  |  |  |  |  |  |  |
| --- | --- | --- | --- | --- | --- | --- |
| **Trait** | **WI** | **CNN** | **CNN(WI3A)** | **Range** | **Mean** | **Heritability** |
| HD (days) | 58.5 | 57.5 | 51.5 | 44-54.5 | 49 | 0.72 |
| PH (cm) | 132 | 128 | 111 | 86-130 | 112 | 0.67 |
| SB (g plant^-1^) | 36 | 36 | 31 | 17-37 | 27 | 0.70 |
| RB (g plant^-1^) | 24 | 23 | 19 | 7.3-26.3 | 17 | 0.64 |
| TB (g plant ^-1^) | 87 | 85 | 89 | 46-96 | 77 | 0.71 |
| KPS | 31 | 25 | 28 | 11-35.6 | 28.4 | 0.70 |
| SWPS (g) | 1.2 | 0.8 | 1.1 | 0.36-1.34 | 1.07 | 0.71 |
| TKW (g) | 37 | 32 | 38 | 27.2-42.3 | 38 | 0.57 |

HD = heading date, PH = plant height, SB = shoot biomass, RB = root biomass, TB = total biomass, TKW = 1000-kernel weight, SWPS = seed weight per spike, KPS = kernels per spike
