# Supplementary material for: Genetic Dissection of Yield and Its Component Traits Using High-Density Composite Map of Wheat Chromosome 3A: Bridging Gaps between QTLs and Underlying Genes
Source: PLoS One. 2013 Jul 24;8(7):e70526. doi: 10.1371/journal.pone.0070526 (PMC3722237; doi:10.1371/journal.pone.0070526)
Supplement: Table S4 — List of QTLs detected on chromosome 3A in different environment-years for seven agronomical traits using 95-RICL 3A mapping population. (DOCX) [file pone.0070526.s007.docx]

**Table S4** List of QTLs detected in different environment-years for seven agronomical traits using 95-RICL 3A mapping population.

|  | | | | | | |
| --- | --- | --- | --- | --- | --- | --- |
| **Field data on seven agronomical traits in 1999 Lincoln** | | | | | | |
| **Trait** | **QTL interval** | **cM** | **LOD** | **R^2^** | **Additive effect** | **Position** |
| HD | *Xpsr1196-Xbg27413* | 2.8 | 2.7 | 12.90 | -0.99 | 64.9 |
|  | Xbg27413-Xtam055 | 3.1 | 2.5 | 13.36 | -1.10 | 67.9 |
| GY | *Xcdo549-Xbcd907* | 3.2 | 2.6 | 14.04 | 0.17 | 2.3 |
|  | *Xbcd907-Xbarc310* | 5.0 | 5.7 | 15.50 | 0.18 | 5.3 |
| TST | *Xbarc106-Xbcd141* | 5.9 | 4.2 | 19.71 | 0.26 | 232.9 |
|  | *Xbcd1555-Xbcd22* | 1.7 | 4.0 | 15.95 | 0.24 | 236.3 |
|  | Xbcd372-Xhbg227 | 15.6 | 3.9 | 21.18 | 0.26 | 243.0 |
| SPSM | *Xbcd907-Xbarc310* | 5.0 | 2.6 | 15.15 | 20.51 | 7.5 |
| **Field data on seven agronomical traits in 2001 Lincoln** | | | | | | |
| **Trait** | **QTL interval** | **cM** | **LOD** | **R^2^** | **Additive effect** | **Position** |
| PH | *Xbarc102 - Xpsr1205* | 16.6 | 2.9 | 15.16 | 1.74 | 342.8 |
| GY | *Xpsp3047-XksuA6* | 7.1 | 3.7 | 15.88 | 0.10 | 157.6 |
|  | *Xcdo638-Xbarc356* | 5.3 | 3.5 | 15.15 | 0.10 | 171.4 |
| TST | *Xpsp3047-XksuA6* | 7.1 | 3.2 | 10.37 | 0.18 | 157.6 |
|  | *Xcdo638-Xbarc356* | 5.3 | 2.7 | 8.54 | 0.17 | 171.4 |
|  | *wPt_2698-wPt_2740* | 7.4 | 2.8 | 8.07 | 0.14 | 307.4 |
| TKW | *wPt_3389-Xgwm497* | 6.2 | 4.6 | 15.02 | 0.38 | 279.4 |
|  | *Xwmc559-Xhbg491* | 1.9 | 4.4 | 14.35 | 0.38 | 284.8 |
|  | *Xhbg491-Xbcd361* | 7.1 | 3.7 | 13.61 | 0.37 | 289 |
|  | *Xbcd361-Xgwm155* | 7.4 | 3.3 | 12.18 | 0.35 | 298.2 |
| SPSM | *Xpsp3047-XksuA6* | 7.1 | 3.9 | 15.37 | 13.67 | 157.6 |
|  | *Xcdo638-Xbarc356* | 5.3 | 4.1 | 17.40 | 20.67 | 171.4 |
|  | *Xgwm666-Xbarc102* | 0.5 | 3.4 | 13.50 | 13.03 | 337.8 |
|  | *Xbarc102-Xpsr1205* | 16.6 | 3.6 | 17.74 | 14.72 | 344.9 |
| KPS | *Xbarc102-Xpsr1205* | 16.6 | 3.0 | 21.60 | -0.92 | 347 |
| **Field data on seven agronomical traits in 2001 Mead** | | | | | | |
| **Trait** | **QTL interval** | **cM** | **LOD** | **R^2^** | **Additive effect** | **Position** |
| PH | *Xcfa2076-Xgwm666* | 6 | 2.5 | 10.90 | 2.21 | 336.1 |
|  | *Xbarc102 - Xpsr1205* | 16.6 | 3.0 | 14.41 | 2.36 | 338.6 |
| GY | *Xcdo638-Xbarc356* | 5.3 | 7.1 | 31.32 | 0.17 | 169.3 |
|  | *Xwmc664-Xbarc67* | 1.1 | 5.2 | 21.80 | 0.14 | 173.9 |
| TKW | *wPt_3041-wPt_4868* | 1.2 | 2.3 | 10.06 | -0.36 | 21.7 |
| SPSM | *Xbcd366-Xbcd927* | 4.6 | 3.5 | 15.11 | 19.84 | 201.4 |
|  | *Xbcd927-Xpsr56* | 5.3 | 3.5 | 15.30 | 19.94 | 204.3 |
| **Field data on seven agronomical traits in 2001 Sidney** | | | | | | |
| **Trait** | **QTL interval** | **cM** | **LOD** | **R^2^** | **Additive effect** | **Position** |
| PH | Xbarc86-Xgwm5 | 9.2 | 3.7 | 19.01 | -1.45 | 156 |
|  | *Xpsp3047-XksuA6* | 7.1 | 4.1 | 18.64 | -1.44 | 157.6 |
|  | *Xcdo638-Xbarc356* | 5.3 | 3.5 | 17.03 | -1.38 | 171.4 |
| TST | *Xcfa2262-Xbcd366* | 8.3 | 3.5 | 13.09 | 0.63 | 189.3 |
|  | *wPt_3389-Xgwm497* | 6.2 | 2.8 | 10.1 | -0.65 | 274 |
|  | *wPt_2698-wPt_2740* | 7.4 | 3.4 | 14.74 | 0.77 | 307.4 |
|  | *Xcfa2076-Xgwm666* | 6.0 | 2.9 | 12.79 | -0.65 | 335.7 |
|  | *Xbarc102 - Xpsr1205* | 16.6 | 3.1 | 18.29 | -0.73 | 340.3 |
| TKW | *wPt_1353-wPt_1655* | 1.2 | 3.1 | 11.94 | -0.96 | 23.8 |
|  | *Xbarc102-Xpsr1205* | 16.6 | 3.5 | 15.64 | -0.76 | 339.1 |
| SPSM | *Xbarc106-Xbcd141* | 5.9 | 3.6 | 18.83 | 16.98 | 232.7 |
|  | *Xbcd22-Xbcd372* | 0.6 | 2.6 | 10.51 | 13.78 | 238.1 |
| KPS | *Xbcd907-Xbarc310* | 5.0 | 4.0 | 15.04 | 0.69 | 5.4 |
|  | *Xwmc11-Xwmc532* | 1.1 | 4.7 | 16.64 | 0.74 | 12.1 |
|  | *Xwmc532-wPt_9303* | 6.9 | 4.8 | 16.68 | 0.73 | 13.8 |
|  | *wPt_3041-wPt_4868* | 1.2 | 4.6 | 16.03 | 0.71 | 21.7 |
|  | *Xwmc428-Xcfa2262* | 2.0 | 3.6 | 12.24 | 0.96 | 187.2 |
|  | *Xbarc106-Xbcd141* | 5.9 | 5.5 | 25.25 | -1.58 | 232.7 |
| **Field data on seven agronomical traits in 2000 Lincoln** | | | | | | |
| **Trait** | **QTL interval** | **cM** | **LOD** | **R^2^** | **Additive effect** | **Position** |
| HD | *wPt_3041-wPt_4868* | 1.2 | 3.2 | 13.5 | 1.28 | 21.4 |
|  | *wPt_1353-wPt_1655* | 1.2 | 2.7 | 11.98 | 1.30 | 24.0 |
|  | *Xbg27413-Xtam055* | 3.1 | 2.8 | 14.11 | -1.14 | 67.9 |
| PH | *Xpsp3047-XksuA6* | 7.1 | 3.7 | 15.36 | -1.74 | 157.9 |
| GY | *Xpsp3047-XksuA6* | 7.1 | 3.3 | 17.53 | 0.10 | 159.8 |
|  | *Xcdo638-Xbarc356* | 5.3 | 2.8 | 13.54 | 0.08 | 169.1 |
| TST | *wPt_3041-wPt_4868* | 1.2 | 2.5 | 9.26 | -0.19 | 21.4 |
|  | *Xhbg491-Xbcd361* | 7.1 | 3.1 | 14.59 | 0.18 | 288.8 |
|  | *Xbcd361-Xgwm155* | 7.4 | 3.2 | 14.22 | 0.18 | 299.6 |
|  | *Xcfa2183-wPt_2967* | 3.6 | 4.9 | 24.26 | 0.27 | 305.6 |
| SPSM | *Xbarc324-Xwmc489* | 1.2 | 2.5 | 9.55 | 19.42 | 176.6 |
| KPS | *Xpsp3047-XksuA6* | 7.1 | 2.6 | 14.28 | 0.90 | 163.9 |
| **Field data on seven agronomical traits in 2000 Mead** | | | | | | |
| **Trait** | **QTL interval** | **cM** | **LOD** | **R^2^** | **Additive effect** | **Position** |
| PH | *Xbarc324-Xwmc489* | 1.2 | 2.8 | 12.44 | -2.99 | 176.6 |
|  | *Xwmc559-Xhbg491* | 1.9 | 3.4 | 12.02 | -2.23 | 285 |
| GY | *Xbcd15-Xcdo395* | 18 | 3.5 | 19.8 | 0.12 | 87.4 |
|  | *XWMC264-Xbarc106* | 1.1 | 3.4 | 13.83 | 0.12 | 227.3 |
|  | *Xbarc106-Xbcd141* | 5.9 | 3.1 | 13.29 | 0.11 | 230.6 |
| SPSM | *Xbarc310-Xbarc321* | 2.6 | 2.3 | 10.12 | 14.09 | 8.3 |
| **Field data on seven agronomical traits in 2000 Sidney** | | | | | | |
| **Trait** | **QTL interval** | **cM** | **LOD** | **R^2^** | **Additive effect** | **Position** |
| PH | *Xtam055-Xbcd15* | 10.8 | 3.1 | 10.39 | -1.33 | 68.7 |
|  | *Xpsp3047-XksuA6* | 7.1 | 2.8 | 9.07 | -1.33 | 157.6 |
| TST | *Xbarc324-Xwmc489* | 1.2 | 5.8 | 23.26 | 0.23 | 177 |
|  | *Xhbg284-Xwmc428* | 5.9 | 6.6 | 28.53 | 0.26 | 183.4 |
|  | *Xcfa2262-Xbcd366* | 8.3 | 5.1 | 27.6 | 0.26 | 193.1 |
| **Field data on three agronomical traits in 2005 Lincoln** | | | | | | |
| **Trait** | **QTL interval** | **cM** | **LOD** | **R^2^** | **Additive effect** | **Position** |
| PH | *Xbarc12-Xwmc11* | 0.5 | 2.9 | 10.93 | -1.73 | 11.9 |
|  | *wPt_3041-wPt_4868* | 1.2 | 3.7 | 13.93 | -1.94 | 21.4 |
|  | *wPt_1655-Xabc171* | 15.1 | 2.6 | 11.2 | -1.73 | 39.1 |
|  | *Xbarc102-Xpsr1205* | 16.6 | 2.5 | 11.64 | 1.92 | 352.5 |
| TST | *Xbarc86-Xgwm5* | 9.2 | 2.5 | 11.26 | 0.48 | 150 |
|  | *XksuA6-Xcdo638* | 2.4 | 3.6 | 14.07 | 0.61 | 165 |
| **Field data on seven agronomical traits in 2005 Mead** | | | | | | |
| **Trait** | **QTL interval** | **cM** | **LOD** | **R^2^** | **Additive effect** | **Position** |
| PH | *Xpsp3047-XksuA6* | 7.1 | 4.4 | 14.5 | -1.52 | 157.6 |
|  | *Xcdo638-Xbarc356* | 5.3 | 3.8 | 13.78 | -1.51 | 171.4 |
|  | *Xhbg491-Xbcd361* | 7.1 | 2.7 | 9.38 | -1.09 | 286.9 |
|  | *Xwmc388-Xbarc102* | 0.5 | 3.0 | 9.33 | 1.01 | 338.2 |
| TST | *Xpsp3047-XksuA6* | 7.1 | 3.5 | 12.97 | 0.44 | 157.6 |
|  | *Xcdo638-Xbarc356* | 5.3 | 2.9 | 11.58 | 0.42 | 171.4 |
| SPSM | *Xcdo395-Xgwm218* | 8.2 | 2.7 | 12.47 | 25.18 | 99.7 |
|  | *Xcfa2183-wPt_2698* | 3.6 | 3.4 | 12.69 | 21.09 | 304.0 |
|  | *wPt_2967-wPt_2740* | 7.4 | 3.4 | 13.05 | 32.31 | 307.4 |
| KPS | *Xbarc324-Xwmc489* | 1.2 | 2.5 | 10.22 | 0.74 | 177.2 |
|  | *Xwmc264-Xbarc106* | 1.1 | 4.1 | 21.27 | -1.14 | 227.3 |
|  | Xbarc106-Xbcd141 | 5.9 | 5.3 | 23.07 | -1.25 | 229.0 |
| TKW | *Xwmc489-Xstm99* | 2.5 | 3.0 | 12.25 | -0.84 | 178.5 |
|  | *Xbcd366-Xbcd927* | 4.6 | 3.4 | 13.45 | -0.87 | 197.7 |
|  | *Xbcd927-Xpsr56* | 5.3 | 2.5 | 11.21 | -0.78 | 204.3 |
| **Field data on seven agronomical traits in 2005 Sidney** | | | | | | |
| **Trait** | **QTL interval** | **cM** | **LOD** | **R^2^** | **Additive effect** | **Position** |
| PH | *Xpsp3047-XksuA6* | 7.1 | 7.19 | 24.43 | -2.15 | 157.6 |
|  | *Xbarc356-Xwmc664* | 1.1 | 8.0 | 23.88 | -2.13 | 172.6 |
|  | *Xbarc67-Xbarc324* | 2.2 | 8.1 | 24.34 | -2.15 | 174.8 |
|  | *Xwmc489-Xstm99* | 2.5 | 6.4 | 21.24 | -1.97 | 180 |
|  | *Xhbg284-Xwmc428* | 5.9 | 6 | 20.47 | -1.94 | 181.5 |
| TST | *Xbcd372-Xhbg227* | 15.6 | 2.5 | 15.54 | 0.41 | 238.5 |
| TKW | *Xbarc324-Xwmc489* | 1.2 | 3.3 | 12.15 | -0.61 | 177 |
|  | *Xwmc489-Xstm99* | 2.5 | 2.5 | 11.1 | -0.55 | 180 |
|  | *wPt_2740-Xcfa2076* | 17.7 | 4.0 | 26.82 | 1.01 | 314.6 |
| **Field data on seven agronomical traits in 2006 Lincoln** | | | | | | |
| **Trait** | **QTL interval** | **cM** | **LOD** | **R^2^** | **Additive effect** | **Position** |
| PH | *wPt_9303-wPt_2253* | 1.3 | 4.6 | 16.02 | -1.71 | 20.3 |
|  | *wPt_4868-wPt_0302* | 0.6 | 2.7 | 10.06 | -1.19 | 22.9 |
|  | *wPt_2938-Xabc171* | 15.1 | 3.6 | 13.07 | -1.44 | 25.1 |
|  | *Xhbg227-wPt_3816* | 19 | 4.0 | 22.83 | -1.60 | 266.3 |
|  | *Xgwm497-Xcfa2193* | 2.5 | 4.7 | 17.13 | -1.56 | 282 |
|  | *Xhbg491-Xbcd361* | 7.1 | 3.6 | 15.42 | -1.49 | 291 |
| TST | *Xbcd366-Xbcd927* | 4.6 | 4.7 | 18.16 | 0.21 | 197.6 |
| SPSM | *Xbarc324-Xwmc489* | 1.2 | 5.1 | 19.95 | 26.70 | 177 |
|  | *Xwmc489-Xstm99* | 2.5 | 3.7 | 16.48 | 24.30 | 180 |
|  | *Xhbg284-Xwmc428* | 5.9 | 5.6 | 24.75 | 31.20 | 185.3 |
|  | *Xhbg227-wPt_1562* | 19 | 2.5 | 21.39 | -30.81 | 268.1 |
|  | *Xgwm497-Xcfa2193* | 2.5 | 3.6 | 12.89 | -25.53 | 282 |
|  | *Xcfa2076-Xgwm666* | 6.0 | 2.7 | 9.17 | 18.26 | 332.3 |
| KPS | *Xbcd927-Xpsr56* | 5.3 | 2.4 | 10.36 | -0.63 | 202.1 |
| **Field data on seven agronomical traits in 2006 Mead** | | | | | | |
| **Trait** | **QTL interval** | **cM** | **LOD** | **R^2^** | **Additive effect** | **Position** |
| GY | *wPt_3389-Xgwm497* | 6.2 | 3.6 | 17.01 | 95.59 | 274 |
| TST | *Xpsp3047-XksuA6* | 7.1 | 3.0 | 10.86 | 0.29 | 157.6 |
|  | *Xbarc106-Xbcd141* | 5..9 | 3.1 | 21.32 | 0.66 | 230.6 |
|  | *Xhbg491-Xbcd361* | 7.1 | 2.9 | 10.45 | 0.21 | 286.9 |
|  | *Xbcd361-Xgwm155* | 7.4 | 3.1 | 11.21 | 0.21 | 299.8 |
| SPSM | *Xcdo395-Xgwm218* | 8.2 | 2.9 | 11.85 | 16.85 | 103.4 |
|  | *Xcfa2183-wPt_2698* | 3.6 | 2.8 | 9.12 | 12.04 | 303.6 |
| KPS | *wPt_9303-wPt_2253* | 1.3 | 3.4 | 15.04 | 1.32 | 20.4 |
|  | *wPt_3041-wPt_4868* | 1.2 | 3.6 | 15.82 | 1.18 | 21.7 |
|  | *wPt_1353-wPt_1655* | 1.2 | 4.1 | 17.64 | 1.27 | 23.8 |
| TKW | *Xbcd361-Xgwm155* | 7.4 | 2.7 | 11.07 | -0.64 | 294.4 |
| **Field data on seven agronomical traits in 2007 Mead** | | | | | | |
| **Trait** | **QTL interval** | **cM** | **LOD** | **R^2^** | **Additive effect** | **Position** |
| PH | *Xpsp3047-XksuA6* | 7.1 | 2.3 | 11.46 | -1.42 | 158.1 |
| GY | *Xcdo549-Xbcd907* | 3.2 | 2.5 | 10.32 | 148.47 | 2.5 |
|  | *Xbcd907-Xbarc310* | 5.0 | 2.7 | 11.62 | 157.11 | 5.4 |
| TST | *Xbcd361-Xgwm155* | 7.4 | 4.8 | 21.92 | 0.39 | 299.8 |
|  | *Xcfa2076-Xwmc388* | 6.0 | 3.3 | 14.76 | -0.38 | 336.1 |
|  | *Xbarc102-Xpsr1205* | 16.6 | 3.6 | 14.24 | -0.34 | 338.6 |
| SPSM | *Xhbg227-wPt_3816* | 19 | 3.8 | 15.74 | 18.73 | 255.6 |
| **Field data on two agronomical traits in 2008 Pullman** | | | | | | |
| **Trait** | **QTL interval** | **cM** | **LOD** | **R^2^** | **Additive effect** | **Position** |
| *Ps*IL | *Xpsp3047-XksuA6* | 7.1 | 2.7 | 12.61 | -6.92 | 157.9 |
|  | *Xcdo638-Xbarc356* | 5.3 | 3.3 | 15.46 | -7.67 | 171.3 |
|  | *Xbarc67-Xbarc324* | 2.2 | 3.4 | 14.5 | -7.43 | 174.7 |
|  | *Xwmc489-Xstm99* | 2.5 | 2.9 | 12.72 | -6.86 | 178.1 |
|  | *Xhbg284-Xwmc428* | 5.9 | 2.8 | 12.79 | -6.94 | 185.4 |
| **Field data analysis of seven agronomical traits 2001 joint analysis** | | | | | | |
| **Trait** | **QTL interval** | **cM** | **LOD** | **R^2^** | **Additive effect** | **Position** |
| PH | *Xpsp3047-XksuA6* | 7.1 | 2.5 | 8.75 | -1.18 | 157.9 |
|  | *Xbarc102-Xpsr1205* | 16.6 | 3.1 | 15.62 | 1.64 | 340.9 |
| GY | *Xpsp3047-XksuA6* | 7.1 | 8.1 | 30.5 | 0.09 | 159.8 |
|  | *Xcdo638-Xbarc356* | 5.3 | 9.6 | 33.98 | 0.09 | 169.1 |
|  | *Xwmc664-Xbarc67* | 1.1 | 8.3 | 29.24 | 0.08 | 173.6 |
| TST | *Xpsp3047-XksuA6* | 7.1 | 3.1 | 11.16 | 0.18 | 157.9 |
|  | *barc1044-Xbarc324* | 2.1 | 3.7 | 12.99 | 0.20 | 174.8 |
|  | *Xhbg284-Xwmc428* | 5.9 | 3.1 | 12.09 | 0.20 | 183.4 |
|  | *Xcfa2183-wPt_2698* | 3.6 | 3.1 | 11.85 | 0.23 | 305.6 |
| TKW | *wPt_9303-wPt_2253* | 1.3 | 2.8 | 16 | -0.40 | 20.3 |
|  | *wPt_3041-wPt_4868* | 1.2 | 3.4 | 13.01 | -0.36 | 21.4 |
|  | *wPt_9928-wPt_1353* | 0.6 | 4.2 | 16 | -0.40 | 23.2 |
| SPSM | *Xgwm5-Xpsp3047* | 0.5 | 4 | 14.81 | 15.36 | 156.8 |
|  | *Xpsp3047-XksuA6* | 7.1 | 3.6 | 14.28 | 14.10 | 159.8 |
|  | *Xcdo638-Xbarc356* | 5.3 | 3.1 | 11.31 | 10.18 | 171.4 |
|  | *Xbcd1555-Xbcd22* | 1.7 | 2.4 | 7.45 | 9.68 | 236.3 |
|  | *Xcfa2076-wmc388* | 6 | 3.5 | 13.68 | 11.00 | 336.4 |
|  | *Xbarc102-Xpsr1205* | 16.6 | 3.2 | 13.33 | 10.13 | 338.6 |
| KPS | *Xbcd141-Xbcd1555* | 1.6 | 3.3 | 11.57 | -0.70 | 234.8 |
| **Field data analysis of seven agronomical traits 2000 joint analysis** | | | | | | |
| **Trait** | **QTL interval** | **cM** | **LOD** | **R^2^** | **Additive effect** | **Position** |
| PH | *Xbarc310-Xbarc57* | 2.6 | 2.5 | 7.64 | 1.55 | 8.3 |
|  | *Xtam055-Xbcd15* | 10.8 | 3.7 | 12.09 | -1.92 | 68.8 |
|  | *Xpsp3047-XksuA6* | 7.1 | 4.2 | 14.05 | -1.18 | 157.6 |
|  | *Xcdo638-Xbarc356* | 5.3 | 3.4 | 12.59 | -1.12 | 171.4 |
| GY | *Xbarc310-Xbarc57* | 2.6 | 3.4 | 10.98 | 0.05 | 8.3 |
|  | *Xwmc11-Xwmc532* | 1.1 | 3.3 | 10.68 | 0.05 | 11.9 |
|  | *wPt_9303-wPt_2253* | 1.3 | 3.2 | 9.36 | 0.05 | 20.2 |
|  | *wPt_7890-wPt_5486* | 1.2 | 2.9 | 9.36 | 0.05 | 23.8 |
|  | *Xbcd15-Xcdo395* | 18 | 3.1 | 11.73 | 0.05 | 79.4 |
|  | *Xpsp3047-XksuA6* | 7.1 | 3.1 | 15.34 | 0.07 | 159.8 |
|  | *Xcdo638-Xbarc356* | 5.3 | 2.7 | 13.42 | 0.07 | 171.1 |
|  | *Xbarc67-Xbarc324* | 2.2 | 3.5 | 13.25 | 0.08 | 174.7 |
| TST | *Xcfa2183-wPt_2698* | 3.6 | 3.3 | 11.77 | 0.27 | 303.3 |
| TKW | *wPt_3041-wPt_4868* | 1.2 | 3.7 | 15.46 | -0.57 | 21.4 |
|  | *Xtam055-Xbcd15* | 10.8 | 3.2 | 13.77 | 0.67 | 68.6 |
| SPSM | *Xbarc310-Xbarc57* | 2.6 | 3.2 | 12.15 | 21.57 | 8.3 |
| KPS | *XksuA6-Xcdo638* | 2.4 | 2.5 | 9.65 | 0.32 | 165 |
| **Field data analysis of seven agronomical traits 2005 joint analysis** | | | | | | |
| **Trait** | **QTL interval** | **cM** | **LOD** | **R^2^** | **Additive effect** | **Position** |
| HD | *wPt_3041-wPt_4868* | 1.2 | 2.4 | 9.64 | 0.56 | 21.8 |
|  | *Xcfa2262-Xbcd366* | 8.3 | 2.5 | 10.59 | -0.67 | 189.8 |
| PH | *wPt_3041-wPt_4868* | 1.2 | 4.5 | 13.09 | -1.18 | 21.4 |
|  | *Xpsp3047-XksuA6* | 7.1 | 5.3 | 14.27 | -1.27 | 157.9 |
|  | *Xcdo638-Xbarc356* | 5.3 | 4.6 | 13.16 | -1.24 | 171.4 |
|  | *Xhbg491-Xbcd361* | 7.1 | 3.2 | 8.33 | -1.04 | 286.9 |
|  | *Xbcd361-Xgwm155* | 7.4 | 2.9 | 8.22 | -1.05 | 300 |
|  | *Xbarc102-Xpsr1205* | 16.6 | 4.3 | 16.09 | 1.40 | 346.1 |
| TST | *Xpsp3047-XksuA6* | 7.1 | 4.7 | 17.52 | 0.33 | 161.6 |
|  | *Xcdo638-Xbarc356* | 5.3 | 4.4 | 16.35 | 0.33 | 171.4 |
|  | *Xwmc664-Xbarc67* | 1.1 | 4.3 | 14.51 | 0.30 | 174 |
|  | *Xwmc559-Xhbg491* | 1.9 | 2.3 | 7.05 | 0.21 | 285 |
| SPSM | *Xbcd1555-Xbcd22* | 1.7 | 2.1 | 8.21 | 14.35 | 236.3 |
| KPS | *Xbarc324-Xwmc489* | 1.2 | 2.5 | 10.59 | 0.55 | 176.6 |
| TKW | *Xbarc12-Xwmc11* | 0.5 | 2.6 | 9.31 | -0.45 | 11.6 |
|  | *wPt_4868-wPt_0302* | 0.6 | 3.7 | 12.62 | -0.52 | 22.9 |
|  | *Xbarc324-Xwmc489* | 1.2 | 5.3 | 18.81 | -0.88 | 177 |
|  | *Xcfa2262-Xbcd366* | 8.3 | 3.3 | 11.36 | 0.65 | 189.4 |
| **Field data analysis of seven agronomical traits 2006 joint analysis** | | | | | | |
| **Trait** | **QTL interval** | **cM** | **LOD** | **R^2^** | **Additive effect** | **Position** |
| PH | *Xcdo549-Xbcd907* | 3.2 | 2.9 | 11.82 | -0.73 | 2.3 |
|  | *Xbcd907-Xbarc310* | 5 | 2.8 | 12.18 | -0.75 | 5.3 |
|  | *Xbarc57-Xbarc12* | 0.6 | 5.1 | 19.18 | -0.97 | 11.3 |
|  | *wPt_9303-wPt_2253* | 1.3 | 5 | 18.84 | -0.95 | 20.3 |
|  | *wPt_9928-wPt_7890* | 0.6 | 3.9 | 15.1 | -0.84 | 23.3 |
|  | *wPt_2938-Xabc171* | 15.1 | 4 | 17.13 | -0.89 | 25.4 |
|  | *Xbe49917-Xbe42522* | 8 | 3.9 | 18.35 | -0.93 | 48.2 |
|  | *Xtam055-Xbcd15* | 10.8 | 4.2 | 15.97 | -0.87 | 69 |
| GY | *Xpsp3047-XksuA6* | 7.1 | 2.9 | 13.49 | 145.23 | 159.8 |
|  | *Xcdo638-Xbarc356* | 5.3 | 5 | 22.33 | 115.09 | 171 |
| TST | *Xbarc86-Xgwm5* | 9.2 | 3.6 | 16.5 | 0.51 | 156 |
|  | *Xcdo638-Xbarc356* | 5.3 | 2.9 | 9.71 | 0.13 | 171.4 |
| SPSM | *Xpsp3047-XksuA6* | 7.1 | 6.4 | 26.03 | 20.03 | 159.8 |
|  | *barc1044-Xbarc324* | 2.2 | 7.6 | 25.96 | 20.49 | 174.8 |
|  | *Xwmc489-Xstm99* | 2.5 | 4.8 | 19.98 | 18.56 | 180 |
|  | *Xhbg284-Xwmc428* | 5.9 | 6.2 | 25.67 | 21.55 | 185.3 |
| KPS | *Xcdo549-Xbcd907* | 3.2 | 2.8 | 10.96 | 0.70 | 11.6 |
|  | *wPt_3041-wPt_4868* | 1.2 | 4.2 | 15.23 | 0.82 | 21.4 |
|  | *wPt_1353-wPt_2938* | 1.2 | 4.3 | 15.58 | 0.86 | 24 |
|  | *Xhbg284-Xwmc428* | 5.9 | 2.8 | 10.85 | -0.71 | 185.3 |
|  | *wPt_3389-Xgwm497* | 6.2 | 2.5 | 9.06 | 0.69 | 273.4 |
| TKW | *Xbarc57-Xbarc12* | 0.6 | 5.2 | 23.15 | -0.44 | 11.3 |
|  | *Xwmc11-Xwmc532* | 1.1 | 5.6 | 23.69 | -0.44 | 12.8 |
|  | *wPt_3041-wPt_4868* | 1.2 | 4.4 | 19.22 | -0.39 | 21.8 |

PH = plant height, HD = heading date, TKW = 1000-kernel weight, GY = grain yield, KPSM = kernels/square meter, GVWT = grain volume weight, and KPS = kernels per spike, *Ps*IL = *Pseudocercosporella* induced lodging
